# Predicting Vessel Diameter Changes to Up-Regulate Biphasic Blood Flow During Activation in Realistic Microvascular Networks

**DOI:** 10.3389/fphys.2020.566303

**Published:** 2020-10-16

**Authors:** Robert Epp, Franca Schmid, Bruno Weber, Patrick Jenny

**Affiliations:** ^1^Institute of Fluid Dynamics, ETH Zurich, Zurich, Switzerland; ^2^Institute of Pharmacology and Toxicology, University of Zurich, Zurich, Switzerland

**Keywords:** functional hyperaemia, vessel diameter changes, blood flow, realistic microvascular networks, inverse simulation model, parameter inference, activation

## Abstract

A dense network of blood vessels distributes blood to different regions of the brain. To meet the temporarily and spatially varying energy demand resulting from changes in neuronal activity, the vasculature is able to locally up-regulate the blood supply. However, to which extent diameter changes of different vessel types contribute to the up-regulation, as well as the spatial and temporal characteristics of their changes, are currently unknown. Here, we present a new simulation method, which solves an inverse problem to calculate diameter changes of individual blood vessels needed to achieve predefined blood flow distributions in microvascular networks. This allows us to systematically compare the impact of different vessel types in various regulation scenarios. Moreover, the method offers the advantage that it handles the stochastic nature of blood flow originating from tracking the movement of individual red blood cells. Since the inverse problem is formulated for time-averaged pressures and flow rates, a deterministic approach for calculating the diameter changes is used, which allows us to apply the method for large realistic microvascular networks with high-dimensional parameter spaces. Our results obtained in both artificial and realistic microvascular networks reveal that diameter changes at the level of capillaries enable a very localized regulation of blood flow. In scenarios where only larger vessels, i.e., arterioles, are allowed to adapt, the flow increase cannot be confined to a specific activated region and flow changes spread into neighboring regions. Furthermore, relatively small dilations and constrictions of all vessel types can lead to substantial changes of capillary blood flow distributions. This suggests that small scale regulation is necessary to obtain a localized increase in blood flow.

## 1. Introduction

The brain has the ability to up-regulate blood flow in response to a locally varying energy demand (neurovascular coupling). This mechanism has been observed for more than a century (Mosso, 1881; Roy and Sherrington, 1890) and is the basis of various imaging techniques such as functional magnetic resonance imaging (fMRI) (Ogawa et al., 1990; Kwong et al., 1992). However, the exact signaling pathways between neurons, astrocytes and the vasculature, as well as the underlying vasodynamics, are still poorly understood (Hillman, 2014; Weber, 2015; Schmid et al., 2019a).

It is widely accepted that smooth muscle cells (SMCs) regulate arteriole diameters to change pressure and flow distributions in the microvasculature. Pial arterioles (PAs) either dilate or constrict in response to neuronal activation, depending on their distance to the activation center and as a function of time after stimulus (Devor et al., 2007). Descending arterioles (DAs) are vessels that branch off the PAs and supply the capillary bed with blood over cortical depth. Many studies have confirmed that DAs adapt their diameters during functional hyperaemia (Tian et al., 2010; Lindvere et al., 2013; Hall et al., 2014; Hillman, 2014; Mishra et al., 2016; Kisler et al., 2017). Recently, it has been suggested that capillaries are contractible as well (Hall et al., 2014; Mishra et al., 2016; Kisler et al., 2017; Rungta et al., 2018). As capillaries are the vessels closest to tissue, they would be perfectly suited for regulative purposes. Furthermore, there is evidence that blood flow at the level of capillaries first homogenizes, before its magnitude increases (Gutiérrez-Jiménez et al., 2016; Lee et al., 2016), which further suggests that regulation on the capillary level takes place. However, the precise spatio-temporal response of arterioles and capillaries, and whether changes are initiated passively or actively, are currently under debate (Fernández-Klett et al., 2010; Tian et al., 2010; Hall et al., 2014; Hillman, 2014; Hill et al., 2015; Mishra et al., 2016; Kisler et al., 2017; Rungta et al., 2018).

The goal of our work is to provide additional insight, by simulating how blood vessels optimally should change their diameters to achieve specific blood flow distributions in the microvasculature. Importantly, our study addresses this topic from a purely fluid dynamical point of view. Thus, we do not take the transcellular signaling into account, and we do not distinguish between active and passive diameter changes. However, by comparing different scenarios where only subsets of vessels can react, we evaluate, on which scales regulation is likely to take place.

Several numerical works investigated how dilations of selected blood vessels affect the flow field (Reichold et al., 2009; Lorthois et al., 2011b; Lorthois and Lauwers, 2012; Schmid et al., 2015). However, unlike these works, here, we consider the corresponding inverse problem and present a new simulation method which is capable of predicting how individual diameters need to change to achieve desired flow distributions in the brain vasculature. This is done by calculating the sensitivities of time-averaged blood flow distributions with regard to vascular diameter changes and subsequently, adapting the vessel diameters to achieve a desired flow increase. Since we are using a deterministic approach to solve the inverse problem, our method has the unique feature that diameter changes of very large microvascular networks (MVNs) can be predicted at relatively small computational cost. Moreover, our method considers the biphasic nature of blood and deals with the stochastic impact of individual red blood cells (RBCs) on flow resistance (Schmid et al., 2019b). To the best of our knowledge, this is the first numerical work which solves an inverse problem to calculate diameter changes for different activation scenarios in large MVNs. Furthermore, our method can be easily adapted for being used in various data assimilation applications, i.e., to infer simulation parameters such as boundary conditions or to reduce overall modeling uncertainties based on sparse experimental measurements.

Several other methods have been proposed for estimating modeling parameters or boundary conditions in MVNs. Recently, a Bayesian framework was presented to infer boundary conditions based on experimental measurements (Rasmussen et al., 2017) and to tune modeling parameters of an empirical phase separation law (Rasmussen et al., 2018). This Bayesian model automatically treats uncertainties of model parameters and experimental data. However, computational cost can be high for large parameter spaces with thousands of uncertainties. Other works estimated boundary pressure values by solving a weighted least squares problem based on experimental blood flow measurements (Sunwoo et al., 2011; Bollu et al., 2018) and literature data of typical distributions of wall shear stresses and pressures (Fry et al., 2012), or based on particle swarm optimization (Pan et al., 2014).

In the following, we first derive our numerical method as generally as possible for arbitrarily chosen parameters. Subsequently, the parameter space will be restricted to only include diameter changes of a subset of blood vessels and the specific modeling equations will be presented. The capabilities of the method are then demonstrated in a simulation study conducted in small hexagonal and large realistic networks from the mouse cerebral cortex. This study improves our understanding on the impact of vessel diameter changes on the regulation of blood flow and contributes to answer the two following questions: (1) Which vessel types are most relevant for a local regulation and (2) how large are the required diameter changes of individual blood vessels.

## 2. Materials and Methods

Our simulation model consists of two main components: The first is a blood flow simulation framework for MVNs and the second is the corresponding inverse model to predict diameter changes necessary to achieve a predefined flow increase in selected blood vessels. In the following, we describe the two components in detail and introduce a solution algorithm for the overall method.

### 2.1. Network Representation of the Vasculature

**Figure 1:**
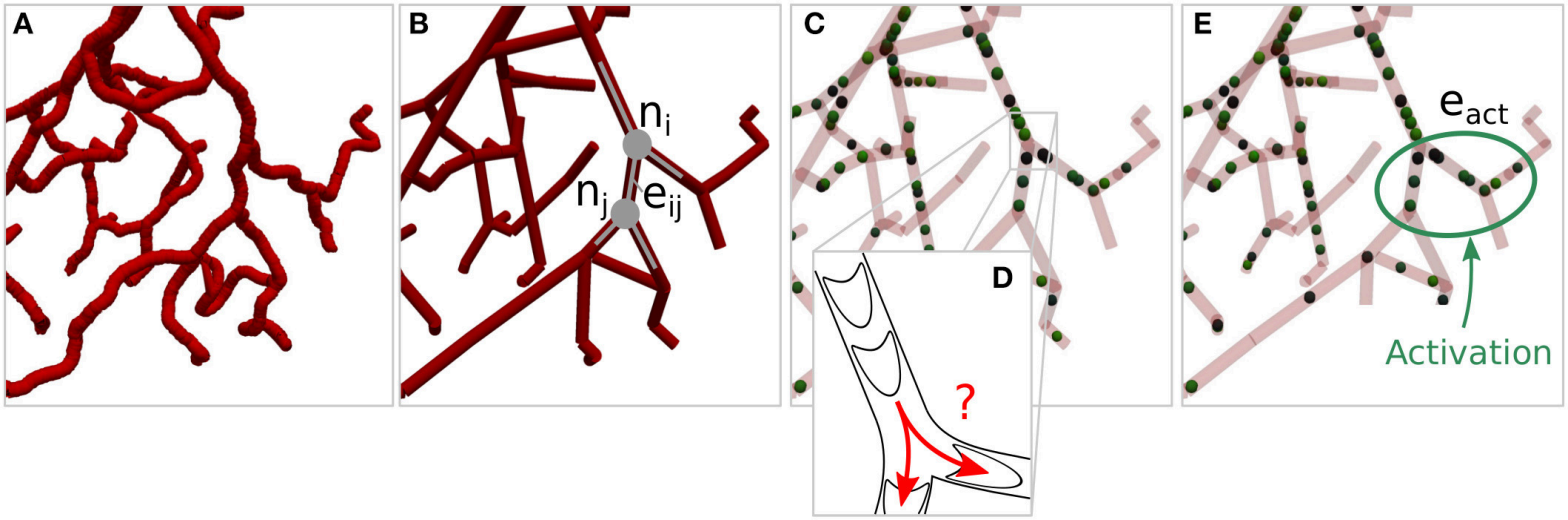
Network representation of a realistic microvascular network and visualization of the tracking of individual red blood cells. **(A)** Tortuous structure of a realistic microvascular network. **(B)** Corresponding network consisting of nodes and edges. The blood vessels are represented by straight pipes. **(C)** Individual red blood cells that follow the flow field and dynamically affect the flow resistance in the network. **(D)** Schematic of red blood cells squeezing through the vessels and bifurcation rule. **(E)** Example of a set of activated edges in which the average perfusion will be increased, i.e., where a target flow rate is prescribed, in response to diameter adaptations.

The microvasculature consists of a dense network of highly interconnected blood vessels (Weber et al., 2008; Blinder et al., 2013). Due to the small diameter scales, the Reynolds and Womersley numbers are small and the complex structure can be modeled by a network consisting of *N*_*e*_ edges representing individual blood vessels and *N*_*n*_ nodes representing connections or intersections of two or more blood vessels. An edge between two nodes *n*_*i*_ ∈ *n* and *n*_*j*_ ∈ *n* is denoted as *e*_*ij*_ ∈ *e*, where *n* and *e* are the sets of all nodes and edges of the network. Attributes of the network corresponding to node *n*_*i*_ and edge *e*_*ij*_ are referred to as *a*_*i*_ and *a*_*ij*_, respectively, where *a* represents the different node and edge parameters as summarized in Table 1. Each individual blood vessel is modeled as a straight pipe with a constant diameter that results in the same flow resistance and length as the corresponding real tortuous vessel. An example of the network representation of a realistic microvascular network is given in Figures 1A,B.

**Table 1:** Node and edge attributes *a* of the network.

**Node attributes**		**Edge attributes**	
Pressure	*p*	Diameter	*d*
Source / sink terms	*b*	Length	*l*
		Number of RBCs	*n*_*rbc*_
		Tube haematocrit	*H*_*t*_
		Discharge haematocrit	*H*_*d*_
		Relative apparent viscosity	μ_*rel*_
		Transmissibility	*T*
		Flow rate	*q*
		Flow direction	*dir*
		Red blood cell velocity	*v*_*rbc*_

### 2.2. Blood Flow Simulation Model

The blood flow simulation model is based on the one previously presented by Schmid et al. (2015, 2017). Here, only the key model concepts are reviewed to briefly introduce the nomenclature. Blood is considered a biphasic fluid consisting of blood plasma and individual RBCs. In every time step [*t*] the flow rate $q_{ij}^{\left[t\right]}$ in edge *e*_*ij*_ is computed by Poiseuille's law, i.e.,

(1) $$\begin{array}{l} q_{ij}^{\left[t\right]}=T_{ij}^{\left[t\right]}\left(p_{i}^{\left[t\right]}-p_{j}^{\left[t\right]}\right), \end{array}$$

where $p_{i}^{\left[t\right]}$ and $p_{j}^{\left[t\right]}$ are the pressures at nodes *n*_*i*_ and *n*_*j*_, and $T_{ij}^{\left[t\right]}$ is the effective transmissibility at time [*t*]. The effective transmissibility is the inverse of the flow resistance, i.e.,

(2) $$\begin{array}{l} T_{ij}^{\left[t\right]}=\frac{\pi d_{ij}^{4}}{128l_{ij}\;\mu_{p}\;\mu_{rel,ij}^{\left[t\right]}}, \end{array}$$

where *d*_*ij*_ and *l*_*ij*_ are the diameter and length of the vessel and μ_*p*_ the viscosity of blood plasma. The Fåhraeus-Linquist effect is modeled by a relative apparent viscosity $\mu_{rel,ij}^{\left[t\right]}$ which depends on the tube haematocrit $H_{t,ij}^{\left[t\right]}$ and the vessel diameter, i.e.,

(3) $$\begin{array}{l} \mu_{rel,ij}^{\left[t\right]}=f\left(H_{t,ij}^{\left[t\right]},d_{ij}\right). \end{array}$$

The tube haematocrit is the volume fraction of RBCs in a vessel, i.e.,

(4) $$\begin{array}{l} H_{t,ij}^{\left[t\right]}=\frac{n_{rbc,ij}^{\left[t\right]}V_{rbc}}{d_{ij}^{2}\frac{\pi }{4}l_{ij}}, \end{array}$$

where $n_{rbc,ij}^{\left[t\right]}$ is the number of RBCs in the vessel and *V*_*rbc*_ the volume of an individual cell. The flow balance equation $g_{i}^{\left[t\right]}$ for each node is

(5) $$\begin{array}{l} g_{i}^{\left[t\right]}:=\underset{n_{k}\in N\left(n_{i}\right)}{\sum }q_{ik}^{\left[t\right]}-b_{i}^{\left[t\right]}=0, \end{array}$$

where $b_{i}^{\left[t\right]}$ is a source or sink term and $N\left(n_{i}\right)$ denotes the set of all neighboring nodes to node *n*_*i*_. Note that $b_{i}^{\left[t\right]}=0$ for all nodes except at boundaries, where $b_{i}^{\left[t\right]}$ is used to account for constant pressure boundary conditions. Since the flow rate is conserved in each node, $g_{i}^{\left[t\right]}=0$ at all times. By combining Equations (1) and (5), a system of linear equations is derived, which can be solved for the pressures and flow rates in the entire network. RBCs are tracked individually as they move through the network and dynamically affect the flow field (Schmid et al., 2019b) (Figure 1C). Due to the Fåhraeus effect, the RBC velocity $v_{rbc,ij}^{\left[t\right]}$ is different from the mean plasma velocity, i.e.,

(6) $$\begin{array}{l} v_{rbc,ij}^{\left[t\right]}=\frac{q_{ij}^{\left[t\right]}}{d_{ij}^{2}\frac{\pi }{4}}\;\frac{H_{d,ij}^{\left[t\right]}}{H_{t,ij}^{\left[t\right]}}, \end{array}$$

where $H_{d,ij}^{\left[t\right]}$ is the discharge haematocrit. We use equations based on empirical data (Pries et al., 1992) for the ratio $H_{d,ij}^{\left[t\right]}/H_{t,ij}^{\left[t\right]}$ of Equation (6) and for calculating $\mu_{rel,ij}^{\left[t\right]}$ in Equation (3). Similarly to Schmid et al. (2017), the RBCs are moved in the network during a constant global time step Δ*t* based on the current $v_{rbc,ij}^{\left[t\right]}$. At divergent bifurcations (Figure 1D), RBCs are assigned to downstream vessels by sampling from a probability function, which was extracted from a bifurcation rule based on experimental data (Pries and Secomb, 2005), i.e.,

(7) $$\begin{array}{l} Prob\left(e_{ij}\right)=FQ_{RBC_{ij}}=f\left(FQ_{B_{ij}},H_{d,parent},D_{parent},D_{ij},D_{ik}\right). \end{array}$$

Here, *Prob*(*e*_*ij*_) is the probability that a RBC enters daughter vessel *e*_*ij*_, *FQ*_*RB*_*C*__*ij*__ and *FQ*_*B*_*ij*__ are fractional RBC flux and blood flow rate in daughter vessel *e*_*ij*_, *H*_*d, parent*_ is the discharge haematocrit in the parent vessel and *D*_*parent*_, *D*_*ij*_ and *D*_*ik*_ are the diameters of the parent and the two daughter vessels, respectively. Compared to Schmid et al. (2015, 2017, 2019b), Equation (7) was used for the entire network and no threshold diameter was introduced, below which RBCs follow the path of the largest pressure force. Note that especially for time averaged simulations on the network scale, the difference between the formulation in Schmid et al. (2015, 2017, 2019b) and in the current work is small (Figures S1, S2). For studies focusing on local effects at single capillary level, we recommend using the formulation by Schmid et al. (2015, 2017, 2019b).

### 2.3. Inverse Model to Infer Parameters of MVNs Based on Time Averaged Blood Flow Characteristics

The blood flow simulation model depends on various modeling parameters such as the diameters and lengths of individual vessels, the bifurcation rule, coefficients that specify the law for calculating the flow resistance and boundary conditions at in- and outflows. In the previous section, we described how a unique solution for the pressure and flow rate distribution is obtained for a fixed set of parameters at each time step. Here, we want to solve the corresponding inverse problem to investigate the influence of parameter changes on the resulting flow field. One possibility to investigate the impact of parameter changes on the flow field is to deflect individual or multiple parameters, e.g., vessel diameters, and compare the resulting flow fields with each other (Reichold et al., 2009; Lorthois et al., 2011b; Lorthois and Lauwers, 2012; Schmid et al., 2015). However, this can be computationally expensive for large parameter spaces. Furthermore, solutions that require a combined change of multiple parameters are difficult to obtain.

In the following, we describe an iterative approach to solve this inverse problem, i.e., to compute possible values for parameters which are required to achieve desired flow or pressure distributions in the network. Due to the tracking of individual RBCs and the stochastic bifurcation rule, $q_{ij}^{\left[t\right]}$ and $p_{i}^{\left[t\right]}$ can be highly unsteady. Since we aim to change the overall flow characteristics in the network, our method is formulated for statistically stationary quantities. The statistically stationary blood flow rate ${\langle q_{ij}\rangle }^{\left(\nu \right)}$ at iteration step (ν) is obtained by averaging over $N_{\Delta t}^{\left(\nu \right)}$ time steps, i.e.,

(8) $$\begin{array}{l} {\langle q_{ij}\rangle }^{\left(\nu \right)}:=\frac{1}{N_{\Delta t}^{\left(\nu \right)}}\overset{N_{\Delta t}^{\left(\nu \right)}}{\underset{t=1}{\sum }}q_{ij}^{\left[t\right]}. \end{array}$$

Similarly, statistically stationary pressures ${\langle p_{i}\rangle }^{\left(\nu \right)}$ and source terms ${\langle b_{i}\rangle }^{\left(\nu \right)}$ are computed. Furthermore, we define a representative transmissibility ${\tilde{T}}_{ij}^{\left(\nu \right)}$ as

(9) $$\begin{array}{l} {\tilde{T}}_{ij}^{\left(\nu \right)}:=\frac{{\langle q_{ij}\rangle }^{\left(\nu \right)}}{{\langle p_{i}\rangle }^{\left(\nu \right)}-{\langle p_{j}\rangle }^{\left(\nu \right)}}. \end{array}$$

In analogy to Equations (1) and (5), we can now find a flow balance equation ${\tilde{g}}_{i}^{\left(\nu \right)}$ which is consistent with the time averaged pressures and flow rates at each node, i.e.,

(10) $$\begin{array}{l} {\tilde{g}}_{i}^{\left(\nu \right)}=\underset{n_{k}\in N\left(n_{i}\right)}{\sum }{\tilde{T}}_{ik}^{\left(\nu \right)}\left({\langle p_{i}\rangle }^{\left(\nu \right)}-{\langle p_{k}\rangle }^{\left(\nu \right)}\right)-{\langle b_{i}\rangle }^{\left(\nu \right)}=0. \end{array}$$

In the following, it is convenient to interpret ${\tilde{g}}_{i}^{\left(\nu \right)}$ as the i-th element of a vector ${\tilde{g}}^{\left(\nu \right)}\in {\mathbb{R}}^{N_{n}}$. Hence, Equation (10) can be rewritten in matrix form, i.e.,

(11) $$\begin{array}{l} {\tilde{g}}^{\left(\nu \right)}={\tilde{A}}^{\left(\nu \right)}{\langle p\rangle }^{\left(\nu \right)}-{\langle b\rangle }^{\left(\nu \right)}=0, \end{array}$$

with ${\langle p\rangle }^{\left(\nu \right)}\in {\mathbb{R}}^{N_{n}}$ and ${\langle b\rangle }^{\left(\nu \right)}\in {\mathbb{R}}^{N_{n}}$ being vectors containing the pressures and source terms of all nodes in the network. The system matrix ${\tilde{A}}^{\left(\nu \right)}\in {\mathbb{R}}^{N_{n}\times N_{n}}$ depends on the transmissibilities of the whole network, i.e., the vector ${\tilde{T}}^{\left(\nu \right)}\in {\mathbb{R}}^{N_{e}}$. We postulate that the solutions of our inverse problem are the parameter values that minimize a certain predefined cost function *J*(α^(ν)^), with $\alpha \in {\mathbb{R}}^{N_{\alpha }}$ being the vector containing all *N*_α_ modeling parameters. Here, *J* will be defined as the difference between simulated and target flow distributions. However, for other applications, different definitions for *J* could be considered. A minimum of *J* is calculated iteratively by using a gradient-based approach, i.e.,

(12) $$\begin{array}{l} \alpha^{\left(\nu +1\right)}=\alpha^{\left(\nu \right)}-\gamma {\left(\frac{dJ^{\left(\nu \right)}}{d\alpha^{\left(\nu \right)}}\right)}^{T}, \end{array}$$

where $\frac{dJ^{\left(\nu \right)}}{d\alpha^{\left(\nu \right)}}$ is the gradient or sensitivity of *J*^(ν)^ with regard to α, and γ is a constant weighting factor. Note that the inverse problem is ill-posed and multiple local minima may exist for the cost function. If γ is chosen small enough, Equation (12) finds the local minima of *J* in the vicinity of the initial state α^(0)^. Since we are interested in computing how parameters need to change to alter flow distributions compared to a baseline state, we are confident that finding the solution close to the initial α^(0)^ is well-suited for our study. Furthermore, we will demonstrate how the cost function can be augmented with additional constraints, in a subsequent section of this paper.

Calculating $\frac{dJ^{\left(\nu \right)}}{d\alpha^{\left(\nu \right)}}$ with a conventional method requires $O\left(N_{n}\cdot N_{\alpha }\right)$ finite differences, which leads to a very high computational cost for large *N*_α_. Therefore, we use the adjoint method to calculate the sensitivity, i.e.,

(13) $$\begin{array}{l} \frac{dJ^{\left(\nu \right)}}{d\alpha^{\left(\nu \right)}}=\lambda^{T}\frac{\partial {\tilde{g}}^{\left(\nu \right)}}{\partial \alpha^{\left(\nu \right)}}+\frac{\partial J^{\left(\nu \right)}}{\partial \alpha^{\left(\nu \right)}}, \end{array}$$

where λ is found by solving the adjoint equation

(14) $$\begin{array}{l} {\left(\frac{\partial {\tilde{g}}^{\left(\nu \right)}}{\partial {\langle p\rangle }^{\left(\nu \right)}}\right)}^{T}\lambda =-{\left(\frac{\partial J^{\left(\nu \right)}}{\partial {\langle p\rangle }^{\left(\nu \right)}}\right)}^{T}. \end{array}$$

The computational cost of solving the adjoint equation is comparable to solving Equation (11) once. More details on the theory behind the adjoint method can be found in various textbooks and papers on data assimilation and optimization, e.g., in Asch et al. (2016). Furthermore, the adjoint method for parameter estimation in MVNs is derived in Equations (S1–S3).

### 2.4. Application of the Inverse Model for Calculating Diameter Changes to Reach Predefined Flow Distributions

If we can find the derivatives $\frac{\partial {\tilde{g}}^{\left(\nu \right)}}{\partial \alpha^{\left(\nu \right)}}$ and $\frac{\partial J^{\left(\nu \right)}}{\partial \alpha^{\left(\nu \right)}}$, the above described sensitivity analysis can be applied to arbitrarily chosen parameter vectors α^(ν)^. However, in this study, we are specifically interested in predicting how the diameters of MVNs need to adapt to achieve a predefined flow distribution. Hence, the parameter vector α only consists of the relative diameters, i.e.,

(15) $$\begin{array}{l} \alpha_{ij}^{\left(\nu \right)}:=\frac{d_{ij}^{\left(\nu \right)}}{d_{ij}^{\left(0\right)}}, \end{array}$$

where $d_{ij}^{\left(\nu \right)}$ is the diameter at iteration step (ν) and $d_{ij}^{\left(0\right)}$ is the initial diameter at baseline conditions. Our goal is to simulate specific activation scenarios and therefore, to locally increase the average flow rate, i.e., in the set of activated edges *e*_*act*_ ⊆ *e* (Figure 1E). The length-averaged blood flow in the activated region is

(16) $$\begin{array}{l} {\bar{q}}_{sim}^{\left(\nu \right)}:=\frac{1}{l_{act}}\underset{e_{ij}\in e_{act}}{\sum }l_{ij}\;dir_{ij}^{\left(0\right)}{\langle q_{ij}\rangle }^{\left(\nu \right)}, \end{array}$$

where *l*_*act*_ is the total length of all activated edges

(17) $$\begin{array}{l} l_{act}=\underset{e_{ij}\in e_{act}}{\sum }l_{ij} \end{array}$$

and

(18) $$\begin{array}{l} dir_{ij}^{\left(0\right)}:=\text{sgn}\left({\langle q_{ij}\rangle }^{\left(0\right)}\right) \end{array}$$

denotes the edge flow direction at baseline conditions with the property $dir_{ij}^{\left(0\right)}=-dir_{ji}^{\left(0\right)}$. We define a cost function that aims to minimize the difference between ${\bar{q}}_{sim}^{\left(\nu \right)}$ and a certain target value ${\bar{q}}_{tar}$, i.e.,

(19) $$\begin{array}{l} J^{\left(\nu \right)}\left(\alpha^{\left(\nu \right)},{\langle p\rangle }^{\left(\nu \right)}\right)={\left(\frac{{\bar{q}}_{sim}^{\left(\nu \right)}-{\bar{q}}_{tar}}{{\bar{q}}_{tar}}\right)}^{2}. \end{array}$$

The partial derivatives $\frac{\partial J^{\left(\nu \right)}}{\partial \alpha^{\left(\nu \right)}}$ and $\frac{\partial J^{\left(\nu \right)}}{\partial {\langle p\rangle }^{\left(\nu \right)}}$ in Equations (13) and (14) are calculated analytically, i.e.,

(20) $$\begin{array}{l} \frac{\partial J^{\left(\nu \right)}}{\partial \alpha_{ij}^{\left(\nu \right)}}=\{\begin{array}{ll} \frac{2\;l_{ij}\;dir_{ij}^{\left(0\right)}}{l_{act}}\left(\frac{{\bar{q}}_{sim}^{\left(\nu \right)}-{\bar{q}}_{tar}}{{\bar{q}}_{tar}^{2}}\right)\left({\langle p_{i}\rangle }^{\left(\nu \right)}-{\langle p_{j}\rangle }^{\left(\nu \right)}\right)\frac{\partial {\tilde{T}}_{ij}^{\left(\nu \right)}}{\partial \alpha_{ij}^{\left(\nu \right)}} & ,e_{ij}\in e_{act}\\ 0,e_{ij}\notin e_{act} \end{array} \end{array}$$

and

(21) $$\begin{array}{l} \frac{\partial J^{\left(\nu \right)}}{\partial \langle p_{i}^{\left(\nu \right)}\rangle }=\{\begin{array}{ll} \frac{2}{l_{act}}\left(\frac{{\bar{q}}_{sim}^{\left(\nu \right)}-{\bar{q}}_{tar}}{{\bar{q}}_{tar}^{2}}\right)\underset{n_{k}|n_{k}\in N\left(n_{i}\right)\wedge n_{k}\in n_{act}}{\sum }\left(l_{ik}\;dir_{ik}^{\left(0\right)}{\tilde{T}}_{ik}^{\left(\nu \right)}\right) & ,n_{i}\in n_{act}\\ 0,n_{i}\notin n_{act} \end{array} \end{array}$$

where *n*_*act*_ ⊆ *n* is the set of all nodes that are adjacent to at least one activated edge *e*_*ij*_ ∈ *e*_*act*_. Furthermore, the derivatives of ${\tilde{g}}_{i}^{\left(\nu \right)}$ with regard to α^(ν)^ and 〈*p*〉^(ν)^ are

(22) $$\begin{array}{l} \frac{\partial {\tilde{g}}_{i}^{\left(\nu \right)}}{\partial \alpha_{ij}^{\left(\nu \right)}}=\left({\langle p_{i}\rangle }^{\left(\nu \right)}-{\langle p_{j}\rangle }^{\left(\nu \right)}\right)\frac{\partial {\tilde{T}}_{ij}^{\left(\nu \right)}}{\partial \alpha_{ij}^{\left(\nu \right)}} \end{array}$$

and

(23) $$\begin{array}{l} \frac{\partial {\tilde{g}}_{i}^{\left(\nu \right)}}{\partial {\langle p_{l}\rangle }^{\left(\nu \right)}}=\underset{n_{k}\in N\left(n_{i}\right)}{\sum }{\tilde{T}}_{ik}\left(\delta_{il}-\delta_{kl}\right), \end{array}$$

where δ_*ij*_ is the Kronecker delta. Note, that $\frac{\partial {\tilde{g}}^{\left(\nu \right)}}{\partial {\langle p\rangle }^{\left(\nu \right)}}$ is the Jacobian ${\tilde{A}}^{\left(\nu \right)}$ from Equation (11). The derivative $\frac{\partial {\tilde{T}}_{ij}^{\left(\nu \right)}}{\partial \alpha_{ij}^{\left(\nu \right)}}$ in Equations (20) and (22) is

(24) $$\begin{array}{l} \frac{\partial {\tilde{T}}_{ij}^{\left(\nu \right)}}{\partial \alpha_{ij}^{\left(\nu \right)}}=\frac{\pi }{128l_{ij}\mu_{p}}\frac{\partial }{\partial \alpha_{ij}^{\left(\nu \right)}}\left(\frac{{d_{ij}^{\left(\nu \right)}}^{4}}{{\tilde{\mu }}_{rel,ij}^{\left(\nu \right)}}\right), \end{array}$$

where ${\tilde{\mu }}_{rel}^{\left(\nu \right)}\in {\mathbb{R}}^{N_{e}}$ is a representative relative viscosity, which is, similarly to ${\tilde{T}}^{\left(\nu \right)}$, consistent with the time averaged flow rates and pressures, i.e.,

(25) $$\begin{array}{l} {\tilde{\mu }}_{rel,ij}^{\left(\nu \right)}:=\frac{\pi }{128l_{ij}\mu_{p}}\frac{{d_{ij}^{\left(\nu \right)}}^{4}}{{\tilde{T}}_{ij}^{\left(\nu \right)}}. \end{array}$$

Due to the stochastic nature of the blood flow, an exact analytical derivative for $\frac{\partial {\tilde{\mu }}_{rel}^{\left(\nu \right)}}{\partial \alpha^{\left(\nu \right)}}$ cannot be found. We approximate Equation (24) as

(26) $$\begin{array}{l} \frac{\partial {\tilde{T}}_{ij}^{\left(\nu \right)}}{\partial \alpha_{ij}^{\left(\nu \right)}}\approx \frac{\pi {d_{ij}^{\left(0\right)}}^{4}}{32l_{ij}\;\mu_{p}\;{\tilde{\mu }}_{rel,ij}^{\left(\nu \right)}}{\alpha_{ij}^{\left(\nu \right)}}^{3}, \end{array}$$

and hence assume that $\frac{\partial {\tilde{\mu }}_{rel}^{\left(\nu \right)}}{\partial \alpha^{\left(\nu \right)}}\approx 0$. Although this assumption does not fully apply to all diameters and haematocrit values, we believe it is justified, since the direction of the approximate $\frac{\partial {\tilde{T}}_{ij}^{\left(\nu \right)}}{\partial \alpha_{ij}^{\left(\nu \right)}}$ is always consistent with the exact derivative. Therefore, this simplification primarily affects the convergence rate, and not the overall result of the inverse problem.

Note that the inverse model only requires the actual ${\tilde{\mu }}_{rel,ij}$ field, and not its derivatives (Equation 26). Consequently, it is flexible with respect to the precise formulation of the blood flow model, i.e., it can be combined easily with different bifurcation rules (Figures S1, S2) and it can also be applied for continuous blood flow models (Lorthois et al., 2011a; Safaeian and David, 2013; Gould and Linninger, 2015).

### 2.5. Augmentation of the Cost Function With Secondary Constraints to Obtain Non-ambiguous Solutions

In the previous sections we ignored that *J* can have multiple minima and many solutions for the inverse problem may exist. For the example shown in Figure 1E this means that different combinations of diameter changes of individual blood vessels could achieve the desired flow change in *e*_*act*_. We reduce this ambiguity by augmenting the inverse problem with additional constraints, i.e., by specifically seeking solutions that also minimize flow rate changes outside of *e*_*act*_, if compared to baseline. The modified cost function reads

(27) $$\begin{array}{l} J^{\left(\nu \right)}\left(\alpha^{\left(\nu \right)},{\langle p\rangle }^{\left(\nu \right)}\right)={\underset{\text{I}}{\underset{︸}{\left(\frac{{\bar{q}}_{sim}^{\left(\nu \right)}-{\bar{q}}_{tar}}{{\bar{q}}_{tar}}\right)}}}^{2}+\epsilon \;{\underset{\text{II}}{\underset{︸}{\left(\rho^{\left(\nu \right)}-\rho_{min}\right)}}}^{2}, \end{array}$$

where the first term (I) is identical to Equation (19) and motivated by the primary target, i.e., to reach the flow increase in *e*_*act*_. The additional constraints are included in the second term (II), where ρ^(ν)^ is the Euclidean norm of the flow rate changes outside of *e*_*act*_, i.e.,

(28) $$\begin{array}{l} \rho^{\left(\nu \right)}=\sqrt{\underset{e_{ij}\notin e_{act}}{\sum }{\left(\frac{{\langle q_{ij}\rangle }^{\left(\nu \right)}-{\langle q_{ij}\rangle }^{\left(0\right)}}{{\bar{q}}_{tar}}\right)}^{2}}, \end{array}$$

and ρ_*min*_ is the minimum possible constant value that still enables full convergence of term I in Equation (27). The precise value of ρ_*min*_ is not known initially and has to be determined iteratively. In our implementation we use the ρ^(ν)^ which we would obtain if term II of the cost function was neglected, as an initial value for ρ_*min*_, and subsequently reduce the value until its minimum is reached. In other words, we first converge our solution to the local minima we find with the gradient-based approach (Equation 12) and the cost function without secondary constraints (Equation 19). In a second step, we activate term II of Equation (27) and iteratively reduce ρ_*min*_. This allows us to move along the solution manifold of *J* ≈ 0 given $\tilde{g}=0$, until the minimum ρ_*min*_ is found, i.e., when *J* ≠ 0 given $\tilde{g}=0$, any more. Although it is difficult to prove that we reach the most-suitable solution for this inverse problem, we are confident that with this strategy we obtain the solution which achieves the desired ${\bar{q}}_{tar}$ in *e*∈*e*_*act*_, minimizes the flow rate changes compared to baseline in *e*∉*e*_*act*_ and is in the neighborhood of the initial state α^(0)^. Keep in mind that our goal is to find the solution closest to the initial state and term I approaches ≈ 0 for all cases.

In Equation (27) ϵ is a constant weighting factor, which is chosen such that both terms I and II are of approximately the same order of magnitude. Since the cost function is always converged to a value *J* ≈ 0, the precise value does not change the final result and primarily affects the convergence rate of the method.

Note, that the cost function here is very different to what is used in conventional variational data assimilation algorithms, where usually a weighted sum of both prior knowledge and observations are used (Asch et al., 2016). However, this conventional approach would lead to solutions where the desired target flow rate is not matched exactly, depending on the weighting factor.

For completeness, the partial derivatives of *J* are

(29) $$\begin{array}{l} \frac{\partial J^{\left(\nu \right)}}{\partial \alpha_{ij}^{\left(\nu \right)}}=\{\begin{array}{l} \frac{2\;l_{ij}\;dir_{ij}^{\left(0\right)}}{l_{act}}\frac{{\bar{q}}_{sim}^{\left(\nu \right)}-{\bar{q}}_{tar}}{{\bar{q}}_{tar}^{2}}\left({\langle p_{i}\rangle }^{\left(\nu \right)}-{\langle p_{j}\rangle }^{\left(\nu \right)}\right)\frac{\partial {\tilde{T}}_{ij}^{\left(\nu \right)}}{\partial \alpha_{ij}^{\left(\nu \right)}},e_{ij}\in e_{act}\\ 2\epsilon \;\frac{\rho^{\left(\nu \right)}-\rho_{min}}{\rho^{\left(\nu \right)}}\frac{{\langle q_{ij}\rangle }^{\left(\nu \right)}-{\langle q_{ij}\rangle }^{\left(0\right)}}{{\bar{q}}_{tar}^{2}}\left({\langle p_{i}\rangle }^{\left(\nu \right)}-{\langle p_{j}\rangle }^{\left(\nu \right)}\right)\frac{\partial {\tilde{T}}_{ij}^{\left(\nu \right)}}{\partial \alpha_{ij}^{\left(\nu \right)}},e_{ij}\notin e_{act} \end{array} \end{array}$$

and

(30) $$\begin{array}{l} \frac{\partial J^{\left(\nu \right)}}{\partial \langle p_{i}^{\left(\nu \right)}\rangle }=\{\begin{array}{l} \frac{2}{l_{act}}\left(\frac{{\bar{q}}_{sim}^{\left(\nu \right)}-{\bar{q}}_{tar}}{{\bar{q}}_{tar}^{2}}\right)\underset{n_{k}|n_{k}\in N\left(n_{i}\right)\wedge n_{k}\in n_{act}}{\sum }\left(l_{ik}\;dir_{ik}^{\left(0\right)}{\tilde{T}}_{ik}^{\left(\nu \right)}\right)\\ +2\epsilon \;\frac{\rho^{\left(\nu \right)}-\rho_{min}}{\rho^{\left(\nu \right)}\;{\bar{q}}_{tar}^{2}}\\ \times \underset{n_{k}|n_{k}\in N\left(n_{i}\right)\wedge n_{k}\notin n_{act}}{\sum }\left({\langle q_{ik}\rangle }^{\left(\nu \right)}-{\langle q_{ik}\rangle }^{\left(0\right)}\right){\tilde{T}}_{ik}^{\left(\nu \right)},\;n_{i}\in n_{act}\\ 2\epsilon \;\frac{\rho^{\left(\nu \right)}-\rho_{min}}{\rho^{\left(\nu \right)}\;{\bar{q}}_{tar}^{2}}\underset{n_{k}\in N\left(n_{i}\right)}{\sum }\left({\langle q_{ik}\rangle }^{\left(\nu \right)}-{\langle q_{ik}\rangle }^{\left(0\right)}\right){\tilde{T}}_{ik}^{\left(\nu \right)},n_{i}\notin n_{act} \end{array} \end{array}$$

respectively.

Alternatively, we could also specify different or additional secondary constraints in a similar way, e.g., to aim for the solution that minimizes the diameter changes of the vessels or the variance of all flow rates in the network.

### 2.6. Solution Algorithm

The overall solution algorithm is summarized in Algorithm 1. The term of *J* containing the secondary constraints is initially deactivated, i.e., initially ϵ = 0. The simulation loop is repeated until *J*^(ν)^ is converged below a certain tolerance value *tol* and the minimum ρ_*min*_ has been reached.

**Algorithm 1:** Algorithm for simulating activation scenarios

1: initialize relative diameters α^(0)^ = 1
2: deactivate secondary constraints ϵ = 0
3: ν ← 0
4: **while** simulation is running **do**
5: BLOOD FLOW WITH RBC DYNAMICS
6: **for** each time step $t=1\ldots N_{\Delta t}^{\left(\nu \right)}$ **do**
7: compute *q*^[*t*]^ and *p*^[*t*]^ based on the current RBC distribution (Equations 1–6)
8: move RBCs for a time step Δ*t*
9: compute averages 〈*q*〉^(ν)^ and 〈*p*〉^(ν)^ (Equation 8)
10: compute ρ^(ν)^ (Equation 28)
11: update cost function *J*^(ν)^ (Equation 27)
12: **if** *J*^(ν)^ > *tol* **then**
13: UPDATE RELATIVE DIAMETERS
14: compute ${\tilde{T}}^{n}$, ${\tilde{\mu }}_{rel}^{\left(\nu \right)}$ and $\frac{\partial {\tilde{T}}^{\left(\nu \right)}}{\partial \alpha^{\left(\nu \right)}}$ (Equations 9,25,26)
15: compute $\frac{\partial {\tilde{g}}^{\left(\nu \right)}}{\partial \alpha^{\left(\nu \right)}}$ and $\frac{\partial {\tilde{g}}^{\left(\nu \right)}}{\partial {\langle p\rangle }^{\left(\nu \right)}}$ (Equations 22,23),
16: compute $\frac{\partial J^{\left(\nu \right)}}{\partial \alpha^{\left(\nu \right)}}$ (Equation 29) and $\frac{\partial J^{\left(\nu \right)}}{\partial {\langle p\rangle }^{\left(\nu \right)}}$ (Equation 30)
17: compute sensitivity $\frac{dJ^{\left(\nu \right)}}{d\alpha^{\left(\nu \right)}}$ (Equations 13 and 14)
18: update parameters α^(ν+1)^ (Equation 12)
19: **else**
20: UPDATE SECONDARY CONSTRAINTS
21: **if** ϵ = 0 **then**
22: activate secondary constraints, e.g., ϵ = 1
23: set new $\rho_{min}<\rho^{\left(\nu \right)}$
24: ν ← ν + 1
25: **return** final parameters α^(ν)^

### 2.7. Convergence of the Method and Impact of Secondary Constraints

**Figure 2:**
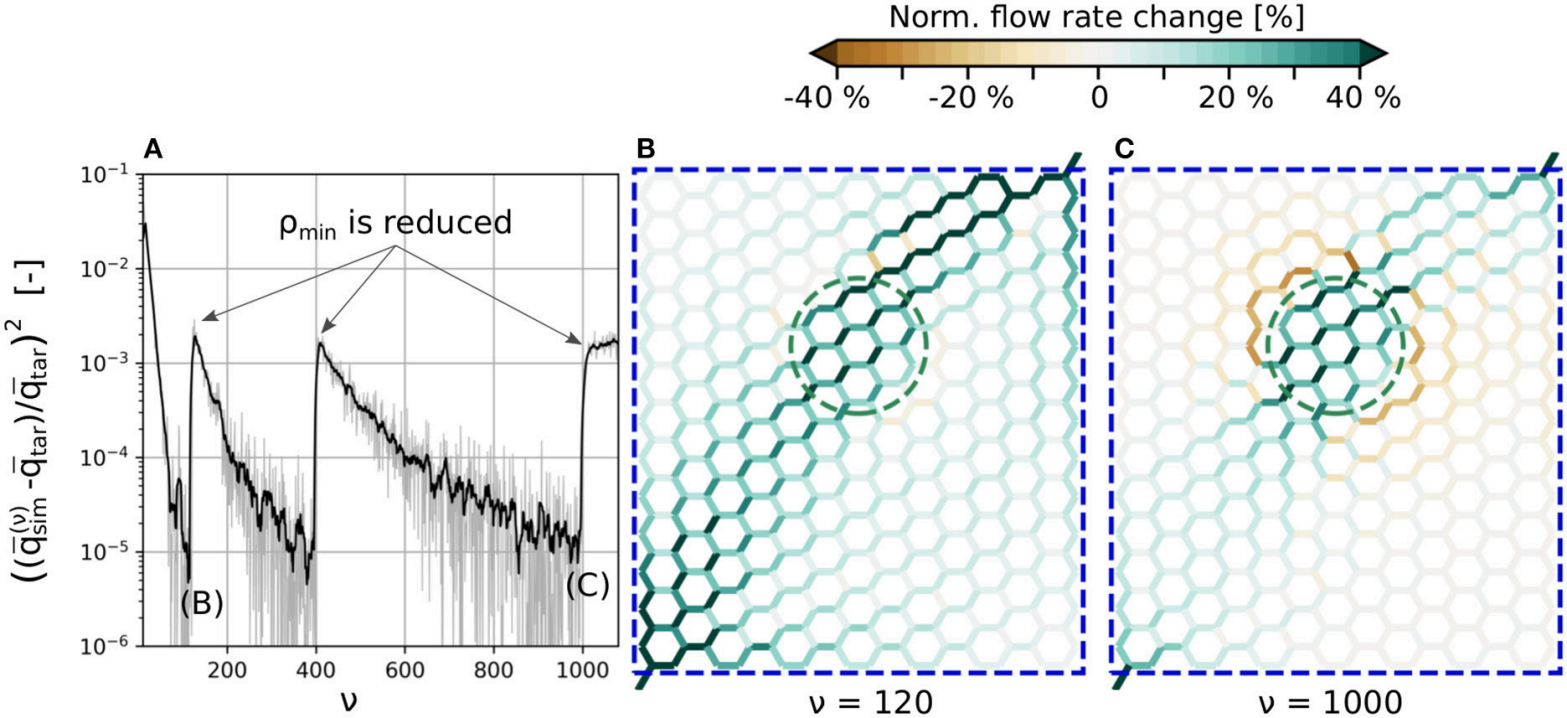
Convergence of the cost function and computed flow rate changes with/without secondary constraints. **(A)** Instantaneous and filtered convergence of primary constraints of *J*. **(B)** Flow rate changes at ν = 120 without secondary constraints. **(C)** Flow rate changes for the final result with the minimum ρ_*min*_ at ν = 1, 000. The average flow rate is increased by 30% in the edges encircled by the green dashed line. All edges within the blue dashed line are allowed to change their diameters to up-regulate flow. The absolute flow rate changes are normalized with the mean baseline flow rate in the activated region, i.e., ${\bar{q}}_{sim}^{\left(0\right)}$. For more information on how the normalized flow rate changes were calculated, and for a visualization of the absolute flow distributions at ν = 0, ν = 120 and ν = 1, 000, see Figure S4.

The typical convergence behavior of term I in Equation (27) is shown in Figure 2A, for a test case in a hexagonal network. The goal was to locally increase the flow rate by 30% in a subset of blood vessels, i.e., in the vessels within the green dashed line shown in Figures 2B,C. The simulation setup and results will be analyzed in more detail in the subsequent section. Here, we will focus on the influence of ρ_*min*_ on the convergence rate and the resulting flow field.

The tracking of individual RBCs and the approximation for calculating $\frac{\partial {\tilde{T}}^{\left(\nu \right)}}{\partial \alpha^{\left(\nu \right)}}$ introduced in Equation (26) result in a noisy convergence of *J*^(ν)^ (gray line). Therefore, a filtered convergence curve (black line) is also shown to better visualize the overall behavior.

The convergence tolerance is *tol* = 10^−5^ and $N_{\Delta t}^{\left(\nu \right)}=500$ time steps are used for time averaging between each iteration step ν. The overall convergence rate depends on γ from Equation (12), which is chosen as large as possible, while the calculated diameter changes still reach their final values without oscillations in almost all blood vessels. Initially, term II of Equation (27) is deactivated (ϵ = 0) until the method converges at ν = 120. Subsequently, term II is activated with $\rho_{min}<\rho^{\left(\nu =119\right)}$. At iteration steps 400 and 1,000 this ρ_*min*_ is further reduced until its minimum value is found. After ν = 1, 000 the method does not converge, since ρ_*min*_ is chosen too small and hence ${\bar{q}}_{tar}$ cannot be reached any more.

Figures 2B,C visualize normalized blood flow changes, compared to baseline at ν = 120 (no secondary constraints) and ν = 1, 000 (final result with minimum ρ_*min*_). Note that the absolute flow rate changes are normalized with the mean baseline flow rate in the activated region, i.e.,

(31) $$\begin{array}{l} \Delta {\langle q_{ij}\rangle }^{\left(\nu \right)}=\frac{dir_{ij}^{\left(0\right)}\left({\langle q_{ij}\rangle }^{\left(\nu \right)}-{\langle q_{ij}\rangle }^{\left(0\right)}\right)}{{\bar{q}}_{sim}^{\left(0\right)}}. \end{array}$$

The primary goal, i.e., the flow increase in the activated region, is achieved in both scenarios. Therefore, both results are solutions for the inverse problem without secondary constraints, i.e., Equation (19). This ambiguity is only resolved with term II of Equation (27) and the minimum ρ_*min*_, which assures that the flow rate changes outside of the activated region are minimized.

The secondary constraint may also impact the inflow rates over the boundaries, if pressures at in- and outflow nodes are kept constant. For example, the flow rate over the boundaries increases by 16.3% for ν = 120, and by 3.9% for ν = 1, 000. Therefore, for ν = 120, the flow increase is primarily achieved by increasing the overall blood supply over the boundaries, i.e., the diameters change in such a way that the overall flow resistance of the network is reduced. For ν = 1, 000, the up-regulation results from diameter changes that not only increase the overall supply, but also internally redistribute the blood flow. This illustrates, that choosing an adequate cost function is a crucial part of the method, since it directly affects the final result.

## 3. Results

**Figure 3:**
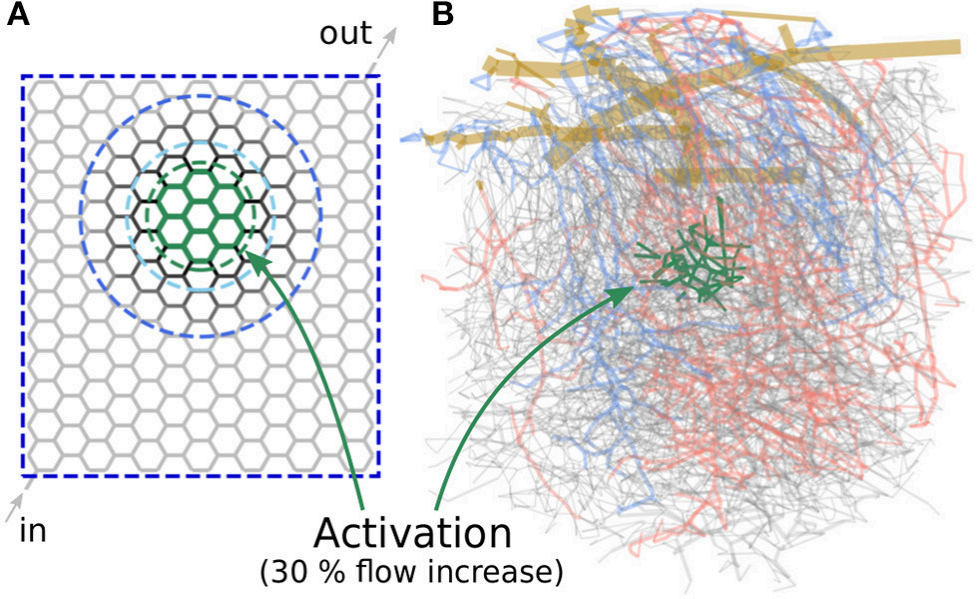
Microvascular networks used in the present study to investigate different activation scenarios. **(A)** Artificial hexagonal network with uniform initial diameters and lengths. The goal is to increase the average blood flow rate by 30% in the green-colored vessels. The vessels which are allowed to change their diameters are encircled by different blue dashed lines, for the three scenarios considered here. **(B)** Realistic microvascular network from the mouse cerebral cortex (network from Blinder et al., 2013). Arterioles are colored red, venules blue and capillaries gray. The goal was to increase the average capillary blood flow rate in the green colored vessels.

Our method allows to investigate various scenarios related to blood flow regulation. We performed simulations in both artificial and large realistic microvascular networks as visualized in Figure 3. The goal was to compute the diameter changes which are required to locally increase the average blood flow rate in a predefined activated region (primary constraint). Outside of the activated region, the flow rates should remain as constant as possible compared to baseline conditions (secondary constraint).

Note, that the scope of our study is not to systematically investigate all possible scenarios, but merely to demonstrate the main capabilities of our method by applying it to different test cases.

### 3.1. Activation in Artificial Hexagonal Networks

Our first study considers an artificial network as shown in Figure 3A. The hexagonal structure is a rough approximation of the highly interconnected capillary bed, where blood vessels are mainly connected through bifurcations. The activated region consists of 30 vessels located approximately in the center of the network (Figure 3A, within the green dashed line) and the target flow rate is ${\bar{q}}_{tar}=1.3\;{\bar{q}}_{sim}^{\left(0\right)}$, which corresponds to a 30% flow increase compared to baseline. In the following, we refer to the vessels within the activated region (*Act*) as *Gen 0* vessels. The adjacent vessels to *Gen 0* are referred to as *Gen 1*, and the next generations of vessels accordingly as *Gen 2* to *Gen 5* vessels.

We compare three scenarios in which different subsets of vessels are allowed to change their diameters to obtain the predefined target flow rate: In the first scenario, all vessels of the entire network are allowed to react. In the other two scenarios, only *Gen 0-5* and *Gen 0-2* vessels can change their diameters, respectively. The vessels which are allowed to adapt are encircled by different blue dashed lines in Figure 3A for each scenario.

At baseline conditions, the vessel diameters are $d_{ij}^{\left(0\right)}=4.5\;\text{µ}\text{m}$ with $\alpha_{ij}^{\left(0\right)}=1$ in the entire network. The vessel lengths are *l*_*ij*_ = 75 µm and constant throughout the simulation. These values are consistent with the mean values of the realistic network from the mouse cerebral cortex shown in Figure 3B (Schmid et al., 2017). Constant pressure boundary conditions are assigned to one in- and one outflow node. Furthermore, a constant inflow tube haematocrit *H*_*t, in*_ = 0.3 is prescribed (Schmid et al., 2019b). Since the computed diameter changes can be large for the scenario where only *Gen 0-2* vessels react, they were restricted to ±15%, i.e., to $0.85<\alpha_{ij}^{\left(\nu \right)}<1.15$, to not obtain results exceeding typical maximal dilations observed in experimental studies (Lindvere et al., 2013; Hall et al., 2014; Rungta et al., 2018). The tolerance for convergence of *J* was set to *tol* = 10^−5^, which corresponds to a discrepancy < 0.5 % between ${\bar{q}}_{sim}^{\left(\nu \right)}$ and ${\bar{q}}_{tar}$.

**Figure 4:**
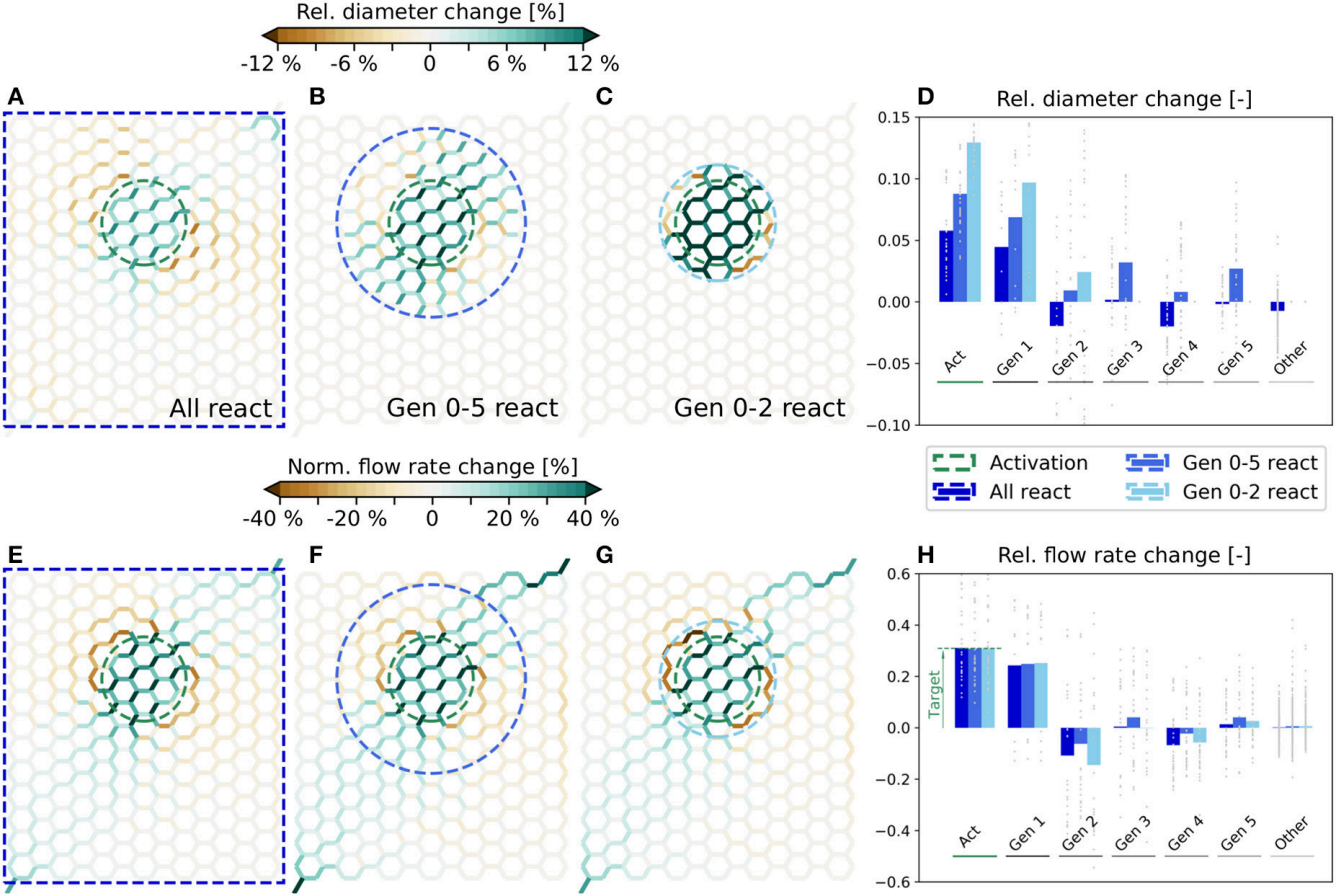
Diameter and flow rate changes for different scenarios in artificial networks. Relative diameter changes of individual vessels for the scenarios where all **(A)**, Gen 0-5 **(B)** and Gen 0-2 **(C)** vessels can react. In each scenario, the blood vessels within the blue dashed lines can adapt. The average flow rate is increased by 30% in the edges within the green dashed circle. **(D)** Relative diameter changes as a function of the vessel generation. Gray dots represent relative changes in individual blood vessels and bars the mean change for each generation. Flow rate changes in individual vessels for the scenarios where all **(E)**, Gen 0-5 **(F)** and Gen 0-2 **(G)** vessels can react. The absolute flow rate changes are normalized with the mean baseline flow rate in the activated region, i.e., ${\bar{q}}_{sim}^{\left(0\right)}$. **(H)** Relative flow rate changes as a function of the vessel generation. Gray dots represent changes in individual vessels with respect to the mean flow rate in the corresponding generation and bars are relative changes of the mean flow rates in each generation.

The simulated relative diameter changes are visualized in Figures 4A–C for all three scenarios. Furthermore, in Figure 4D the average and individual diameter changes for each vessel generation are shown. The corresponding changes of the flow rates are presented in Figures 4E–H. We observe that a very localized flow increase in *Act* is achieved in all three scenarios and that the specified target flow increase of 30% is matched accurately (Figure 4H). Flow rate changes are also observed in the surrounding vessels. While the average flow rate increases in *Gen 1*, it decreases in *Gen 2*. Further away, the average changes are relatively small, i.e., ≲ 5%. However, changes in individual vessels are highly heterogeneous and may differ significantly from the mean values. For example, the flow rate changes range from ≈6−60% in *Act* for all three scenarios (Figure 4H). By comparing Figures 4E–G, we see that the overall characteristics of the flow rate changes are very similar, independent of the number of reacting vessels.

We observe that there are exclusively dilations in *Act*, but a combination of dilations and constrictions outside of *Act* (Figure 4D). The diameter changes are generally small, if all vessels in the network can react, i.e., ≈ 5.8% on average in *Act* (Figure 4A). The changes become larger the fewer vessels are allowed to adapt, i.e., ≈ 8.8% (Figure 4B) and ≈ 12.9% (Figure 4C) on average in *Act*, if only *Gen 0-5* and *Gen 0-2* vessels can react, respectively.

### 3.2. Activation in Realistic Microvascular Networks

The second study investigates different scenarios related to activation in a realistic microvascular network from the mouse cerebral cortex. The network was obtained by Blinder et al. (2013) and the blood vessels were classified as pial arterioles (PA), arterioles (A), capillaries (C), venules (V), and pial venules (PV), based on the network topology and vessel diameters (Blinder et al., 2013; Schmid et al., 2017). In Figure 3B, the colors red, blue, and gray are used to highlight arterioles, venules and capillaries, respectively. The network spans an area of 1.13 mm × 1.15 mm and has a depth of 1.30 mm. It consists of 12,502 edges with a mean capillary diameter of 4.5 µm. For all simulations, a constant time step size of Δ*t* = 0.4 ms is used and the instantaneous flow rates and pressures are averaged over 4 s ($N_{\Delta t}^{\left(\nu \right)}=$10,000) between each iteration step ν. Due to the constant time step size, bifurcation events occur on average in 3.3% of all capillaries at every time step. For more details on the choice of the constant time step size and the averaging time see Figure S3. Constant pressure and haematocrit boundary conditions are assigned to in- and outflow nodes at the level of pial vessels and capillaries (Schmid et al., 2017). While literature data on pressure measurements exists for the larger pial vessels (Shapiro et al., 1971; Harper and Bohlen, 1984; Werber and Heistad, 1984; Hudetz et al., 1987), no such data are available for the capillaries. Therefore, a hierarchical boundary approach was applied by implanting the realistic network into a much larger artificial domain to obtain appropriate boundaries at the capillary level. For more details on the structure of the network, the vessel classification and on how the boundary conditions were obtained we refer to a previous publication of our group (Schmid et al., 2017). The network was taken from the somatosensory cortex and covers parts of the barrel field. Barrels are regions with higher cell density and each of these barrels primarily receives sensory input from one whisker. By stimulating one specific whisker, an increase of neuronal activity and consequently, blood flow, can be observed in the corresponding barrel. Since the locations of the barrels are available (Blinder et al., 2013), we investigate different activation scenarios for one specific barrel. More precisely, the goal of our study is to increase the average capillary blood flow rate by 30% in the most central barrel (*V*_*barrel*_ = 3.9 × 106 fL) of the network, i.e., in the green colored vessels of Figure 3B. Note that the blood vessels defining the activated region are located at the depth of cortical layer IV and consequently, not all vessels of the entire barrel column are included. This is motivated by the observation of laminar differences in vessel topology (Weber et al., 2008; Blinder et al., 2013) and blood flow distributions during baseline and activation (Goense et al., 2012; Schmid et al., 2017; Li et al., 2019). However, the exact temporal and spatial flow patterns on the capillary level are currently unknown and more advanced high-resolution tomographic imaging methods, e.g., Ntziachristos and Razansky (2010); Errico et al. (2015), will be required for its quantification in the future.

We compare five scenarios where only selected vessel types are allowed to dilate or constrict simultaneously. In Table 2, the vessel types, the total number of vessels which are allowed to react at the same time and the corresponding percentage relative to all vessels in the network are listed for each scenario. Furthermore, the fractions of responding vessel volumes and lengths are also given in the table. Due to their close proximity to boundaries, the diameters of pial vessels (PA/PV) are kept constant in all scenarios and only the impact of A, V, and C is considered. It should be noted, that no active regulation of venule diameters has been reported in the literature. However, passive diameter changes may occur based on variations in the pressure field and considerable changes of venous blood volume were observed in MRI-based studies (Chen and Pike, 2009, 2010).

**Table 2:** Investigated scenarios were different vessel types are allowed to react simultaneously.

**Scenario**	**Vessel types**	**Nr. of responding edges**	**Volume responding (%)**	**Length responding (%)**
I	A, V, C	12,427 (99.4 %)	73.7	99.0
II	A, C	10,510 (84.1 %)	52.4	89.3
III	C	9,182 (73.4 %)	35.7	81.5
IV	A, V	2,167 (17.3 %)	27.2	12.1
V	A	1,328 (10.6 %)	16.7	7.8

*For each scenario, the number and percentage of vessels which are allowed to change their diameters, and the corresponding fractional volumes and lengths, are given. Percentage values are calculated relative to all vessels in the entire network, including PAs and PVs*.

#### 3.2.1. Diameter and Blood Flow Rate Changes

**Figure 5:**
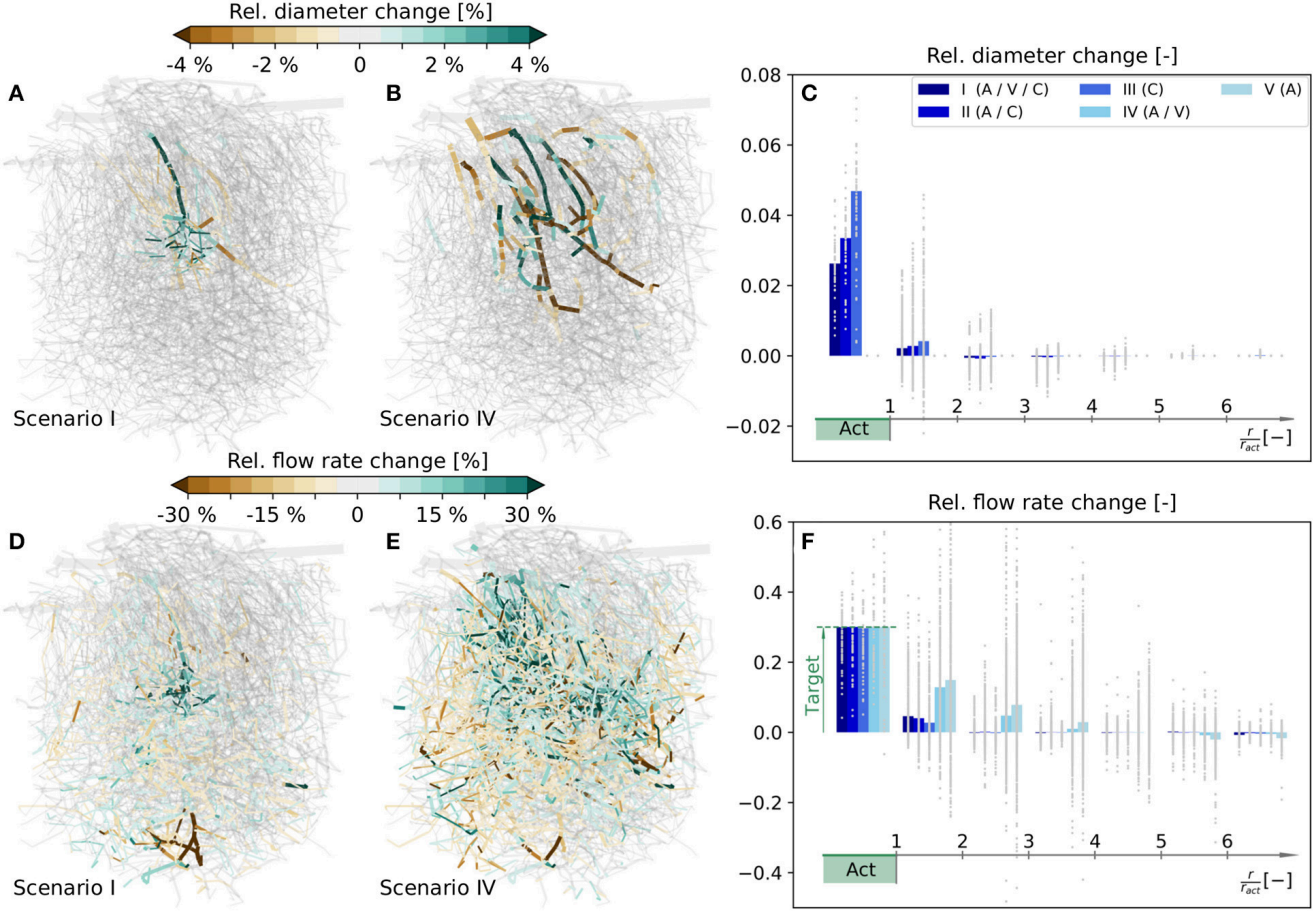
Relative diameter and flow rate changes in a realistic microvascular network, when only selected vessel types are allowed to dilate and constrict. **(A)** Diameter changes, if A, V, and C can react (scenario I). **(B)** Diameter changes, if only A and V can react (scenario IV). **(C)** Capillary diameter changes for scenarios I–V. Results are shown as functions of distance to the barrel center. Gray dots are relative diameter changes in individual averaging cubes, and bars represent mean values obtained by averaging over all cubes within a certain distance to the activation center. **(D–F)** Corresponding relative changes of blood flow rates. In **(F)** the mean blood flow changes in *Act* are calculated based on the values in individual capillaries, i.e., with Equation (16), to demonstrate that the target flow rate increase is reached. For completeness, the relative diameter and flow rate changes for scenarios II, III, and V in the entire network are visualized in Figure S5. Furthermore, absolute flow rate changes normalized with the mean capillary blood flow rate in the activated barrel at baseline, i.e., ${\bar{q}}_{sim}^{\left(0\right)}$, are visualized in Figure S6 for scenarios I–IV.

The simulated relative diameter changes of individual vessels are visualized in Figures 5A,B for scenarios I and IV, respectively, and the corresponding flow rate changes are shown in Figures 5D,E. In the following, our analysis mainly focuses on changes in the capillary bed. For more information on average diameter and blood flow changes of all vessel types, see Tables S1, S2, respectively.

To compare the changes at the capillary level more quantitatively, the network is partitioned into 5355 overlapping averaging cubes of 82 µm side length. For each cube we computed the relative changes of mean capillary diameters and flow rates, and determined the relative distance to the center of the activated barrel, i.e., the polar coordinate *r*/*r*_*act*_, where *r*_*act*_ is the approximate radius of the barrel, i.e., *r*_*act*_ = 98 µm in the current study. The average relative changes in individual cubes are classified into different ranges of distances to the activation center and shown as gray dots in Figures 5C,F. The corresponding bar plots visualize mean changes obtained by averaging over all cubes within a certain distance to the activation center.

Our results reveal that the desired target flow rate in *Act* is reached in all five scenarios (Figure 5F). We observe a very localized flow rate increase in the activated barrel in all scenarios where capillaries are allowed to change their diameters, i.e., in scenarios I–III (Figure 5D). On the contrary, the change is far less local and also spreads into neighboring barrels, if only larger vessels are allowed to react, i.e., in scenarios IV and V (Figure 5E). In scenarios I - IV the flow rates within *Act* exclusively increase. Outside of *Act* there are also regions with a reduction of blood flow (Figure 5F). These regions with reduced blood supply are observed less for scenarios where more blood vessels can react, i.e., in scenarios I - III if compared to IV and V (Figure 5F). As in the artificial network, the flow rate changes outside of *Act* are highly heterogeneous, i.e., a complex pattern of flow increases and decreases is observed (Figures 5D,E). The magnitudes of these changes can be very large, if capillaries are not allowed to change diameters. For example, the increase can reach 60% at distances of *r*/*r*_*act*_ > 2 in individual cubes, which is twice the value of the desired increase in *Act* (Figure 5F). The flow increase is also achieved, if capillaries are the only vessels that change. Hence, from a purely hemodynamic perspective, capillary dilations and constrictions are sufficient to achieve a flow increase of 30% in *Act*.

A closer look at the required diameter changes at the capillary level reveals that they are most pronounced in the range with *r*/*r*_*act*_ < 2 (Figure 5C). Note that in scenarios IV and V the capillary diameter changes are zero by definition. Furthermore, we see that there are only dilations in *Act*, but a combination of dilations and constrictions outside of *Act* (Figure 5C). As expected, individual diameters need to change more, if fewer vessels are allowed to react. For example, the average diameter changes in *Act* are higher in scenarios II and III than in scenario I (Figure 5C). However, the required diameter changes are relatively small for all test cases, especially if we consider that a 5% diameter change corresponds to an absolute change of < 0.5 µm for capillaries. If capillary diameters are kept constant (scenarios IV and V), diameter changes of As and Vs are observed in a large region of the network (Figure 5B).

Since the pressures at boundary nodes are kept constant throughout the simulation, the total blood supply to the network may change based on diameter changes in the network. However, in our study, the changes of total blood flow over the boundaries are relatively small, i.e., < 0.005% for scenarios I - III, ≈ 0.7% for scenario IV and ≈ 1.2% for scenario V.

#### 3.2.2. Tube Haematocrit and RBC Flux Changes

**Figure 6:**
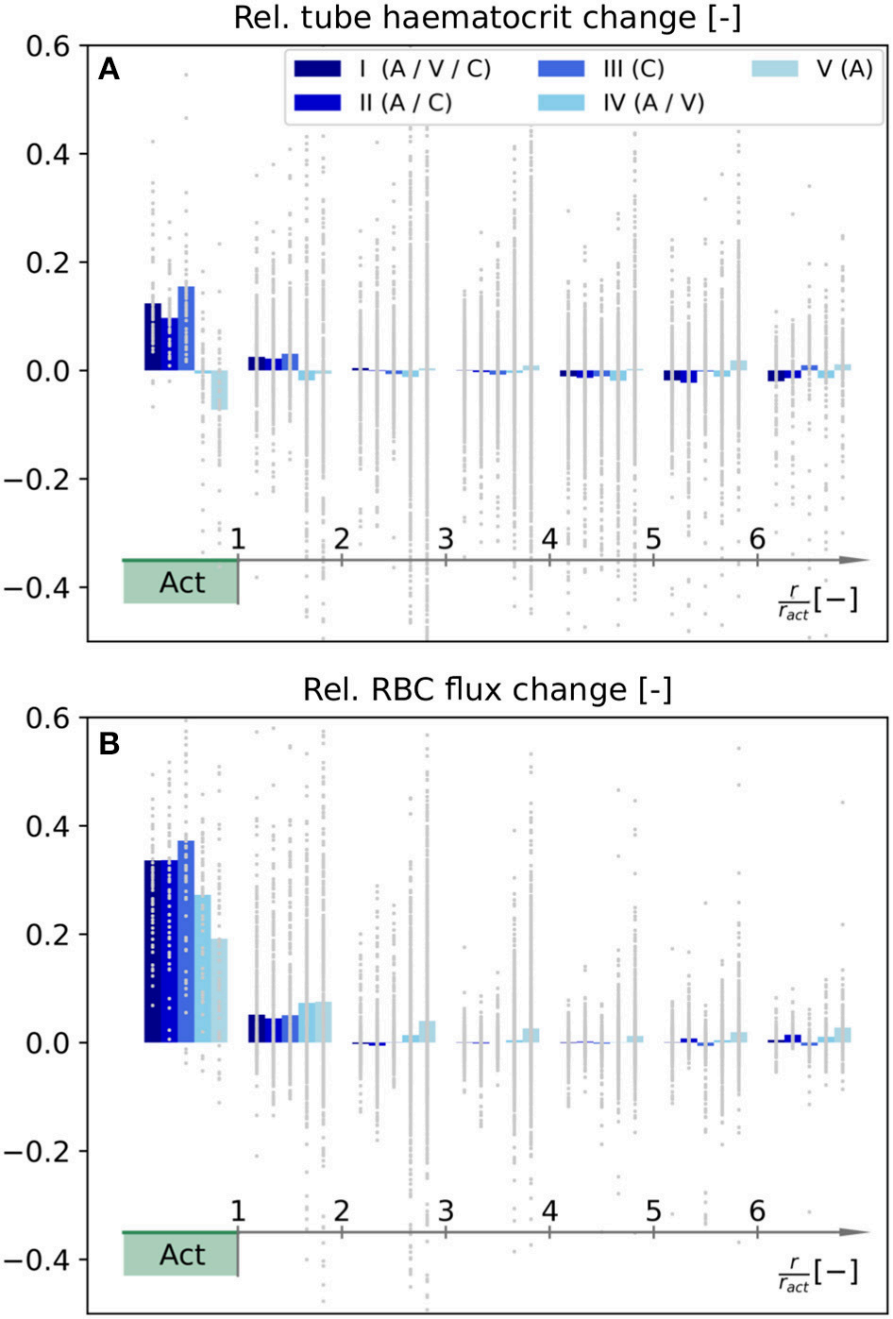
Relative changes of tube haematocrit **(A)** and RBC flux **(B)** as functions of distance to the barrel center. Note that the color legend and the procedure to calculate the relative changes for individual averaging cubes are identical to Figures 5C,F. The RBC fluxes were determined by counting the number of discrete RBCs which enter each blood vessel on average.

In the following, we compare how RBC distributions are affected by the diameter changes presented in Figure 5. Therefore, the average relative changes of tube haematocrit and RBC flux are shown in Figure 6 as functions of distance to the activation center. We want to point out that the goal of our inverse model was to locally increase averaged blood flow rates (Equation 27), and no optimization has been performed with respect to RBC flux and haematocrit. Hence, the relative changes presented in Figure 6 are only passive results, caused by the computed diameter and flow rate changes (Figure 5). Therefore, it is possible that different diameter changes may yield a more optimized result to obtain a localized RBC flux increase in *Act*.

Our results indicate that the tube haematocrit within *Act* increases for scenarios where capillaries adapt, i.e., for scenarios I–III. For the other two scenarios, the average haematocrit remains approximately constant (IV) or even decreases (V). Note that the relative change in haematocrit per averaging cube is highly heterogeneous across the network. This observation is most pronounced for scenarios IV and V (Figure 6A).

Due to phase separation at divergent bifurcations, changes of RBC fluxes are not exactly equivalent to changes of the overall blood flow rate. For scenarios I–III, i.e., with capillary diameter changes, the average RBC flux in *Act* increases more than the corresponding blood flow rate. The opposite trend is observed for scenarios IV and V, where the increase of average RBC flux in *Act* is below the 30%, which was the prescribed blood flow rate increase during activation (Figure 6B).

#### 3.2.3. Consistency of Results Across Barrels

So far we focused on blood flow changes in the most central barrel of the realistic MVN (Figure 3B). To ensure that results are robust across barrels, we repeated our study for two additional barrels as visualized in Figures S7A, S8A. For both barrels, results for the relative changes of diameter, flow rate, tube haematocrit and RBC flux are shown in Figures S7B–J, S8B–J, respectively.

The results for the two additional barrels are comparable to the results in the most central barrel (Figures 5, 6). For example, the blood flow increase of 30% in the activated barrel is reached for all five vessel response scenarios considered. Furthermore, if capillaries are allowed to react (scenarios I - III), the blood flow increase is more confined to the activated region, and the RBC flux increases more than for scenarios where only larger vessels react. Some differences are observed for the diameter distributions of scenario I and the haematocrit distributions of scenarios IV and V. However, due to the highly heterogeneous structure of realistic MVNs, not every barrel is fed by the equal number of vessels of the same types, and therefore, certain differences can be expected. Consequently, we conclude that our observations hold across barrels.

## 4. Discussion

### 4.1. Capillary Diameter Changes Enable a Locally Confined Regulation of Blood Flow

We investigated how dilations and constrictions of different blood vessels influence the local up-regulation of blood flow. Our results indicate that a localized blood flow increase in a specific region of the brain is not possible, if capillaries are rigid and only larger vessels (A+V) change their diameters. In this case, significant flow rate changes also occur outside of the activated region. This can be explained by considering that A+V feed or drain a large number of capillaries and hence, it can be expected that vasodilations and -constrictions of those vessels lead to changes of blood flow distributions in much larger regions of the microvasculature. In contrast, diameter changes at the capillary level lead to a redistribution of blood flow on a much more local scale. Our study suggests that two different mechanisms may be involved in regulating blood flow. On one hand, the larger vessels are responsible for increasing the overall blood supply to the activated region. On the other hand, diameter changes at the capillary level may be necessary to confine the blood flow changes to a particular region, e.g., to a specific barrel of the somatosensory cortex. It is currently difficult to validate our results with experimental data, since diameter changes would have to be measured in many vessels simultaneously. However, our findings are in line with various *in vivo* studies which show that arterioles as well as capillaries change their diameters during activation (Hall et al., 2014; Mishra et al., 2016; Kisler et al., 2017; Rungta et al., 2018).

We want to point out that our simulation framework seems to be a valuable tool to better understand the possible impact of various cell types on regulation. For example, one could think of scenarios where only the vessels which are wrapped by SMCs could change their diameters and compare the results to test cases where also vessels with pericytes can react.

We further observed that the blood flow increase is also achieved, if only capillaries change their diameters and A+V are rigid. It should be noted that this is not necessarily a realistic scenario, since it is known that arterioles dilate *in vivo* (Tian et al., 2010; Lindvere et al., 2013; Hall et al., 2014; Hillman, 2014; Mishra et al., 2016; Kisler et al., 2017). However, it demonstrates that, from a purely hemodynamic point of view, simultaneous dilations and constrictions of multiple capillaries can locally induce a flow increase comparable to diameter changes at the level of A+V. These results are consistent with previous numerical studies which also reported that significant changes of blood flow rates are possible in response to dilations of capillaries only (Lorthois and Lauwers, 2012).

It has been suggested that the location of the largest pressure drop is the optimal place for regulating blood flow (Hall et al., 2014). However, based on our results, it seems likely that the vasodynamic response is complex and not easy to describe in full detail (Lindvere et al., 2013). In the scenarios considered, we observed that the most local regulation is achieved with very heterogeneous and small positive and negative diameter changes.

A previous study of our group shows that the location of the largest pressure drop varies over cortical depth (Schmid et al., 2017). Hence, it should be further investigated if those differences over depth affect the blood flow regulation, since different mechanisms may play a role depending on the exact location of the activated region.

Due to phase separation at divergent bifurcations, RBC flux and blood flow distributions do not change equally in the activated region. This is also observed in our results, where the RBC flux increase in *Act* is larger than the corresponding blood flow change, if Cs can change their diameters. This is in line with previous observations of Schmid et al. (2015, 2019b), which report an increase in RBC flux in response to capillary dilation. For scenarios where only As and Vs adapt, the opposite trend is observed and the RBC flux increase is below the blood flow increase. We suggest that capillary diameter changes are beneficial both for the localized up-regulation of flow and haematocrit. This is of particular interest, since Lücker et al. (2017) recently showed that haematocrit as well as RBC velocity have a large influence on the actual tissue oxygenation. However, as we did not optimize the diameter changes to obtain a prescribed RBC flux, further investigations are necessary to confirm this hypothesis.

### 4.2. Substantial Blood Flow Increase Is Achieved With Relatively Small Dilations and Constrictions

Our results show that a substantial blood flow increase is achieved with relatively small vessel diameter changes. This can be explained, if we consider that the relative flow resistance change of one single vessel is inversely proportional to the fourth power of the relative diameter, i.e.,

(32) $$\begin{array}{l} \frac{R_{ij}^{\left(\nu \right)}-R_{ij}^{\left(0\right)}}{R_{ij}^{\left(0\right)}}\sim \left(\frac{1}{{\alpha_{ij}^{\left(\nu \right)}}^{4}}-1\right). \end{array}$$

For example, a diameter increase of 5% reduces the flow resistance in an individual vessel by 17.7%, if we only consider plasma flow and neglect the impact of RBCs. This is in line with our observation of exclusively dilating vessels in *Act*. The blood supply in *Act* is further increased by dilations of vessels which are located up- or downstream of *Act*. Note that from a purely hemodynamic point of view and in a symmetric network, both up- and downstream diameter changes would have an identical influence on the overall resistance and thus affect the flow in vessels equivalently. Besides dilations, we also observed vasoconstrictions outside of *Act*. These constrictions are responsible for rerouting blood from regions outside of *Act* toward the inside to further increase local perfusion.

Generally, the diameter changes are highly heterogeneous and form a complex interplay of dilations and constrictions. Such heterogeneous responses were also observed in previous *in vivo* studies, where activation induced diameter changes were detected simultaneously in multiple blood vessels (Lindvere et al., 2013).

For all scenarios where capillaries can react (I–III) the computed diameter and flow rate changes are located within or in the closer vicinity of *Act*. If only A+V are allowed to change (IV and V), the changes spread over the entire network. In this case larger microvascular networks would be beneficial to simulate the diameter changes and to better quantify the locality of the regulation.

In our study in hexagonal networks we compared how the number of reacting vessels affect the flow distributions. Although the computed diameter changes vary considerably as a function of the number of reacting vessels, the corresponding flow rate changes show a very similar behavior for all scenarios. Hence, similar flow patterns can be achieved, if either a small number of vessels experience pronounced diameter changes, or if a much larger set of vessels reacts mildly.

Importantly, the computed diameter changes are in a range which is difficult to be detected *in vivo*. For the capillary bed we observe relative changes of approximately 1–6%, which, for an average diameter of 4.5 µm, correspond to absolute changes in the range of 0.05–0.27 µm. This is below the resolution of most experimental methods and has to be kept in mind for *in vivo* experiments.

In our current analysis we assume that each vessel reacts homogeneously over its entire edge length. Due to varying mural cell densities along the vessels, this assumption might not hold *in vivo*, e.g., since it has been observed that capillary pericytes may change diameters locally along individual vessels (Hall et al., 2014). By neglecting the influence of individual RBCs on resistance, our results can easily be transformed to the case where vessels dilate or constrict partially over their length. If only the fraction β_*ij*_ of an edge *e*_*ij*_ is able to react, the relative diameter α_β, *ij*_, with

(33) $$\begin{array}{l} \alpha_{\beta ,ij}\approx {\left(\frac{\beta_{ij}}{1/\alpha_{ij}^{4}-1+\beta_{ij}}\right)}^{1/4}, \end{array}$$

yields the same flow resistance as the case with a relative diameter α_*ij*_ over the entire edge length. For example, entire vessel-length changes of 1 and 5% would correspond to changes of approximately 4 and 36%, if only 25% of the vessel lengths could react.

Recent studies suggest that in response to changes in tissue oxygenation, the deformability of RBCs is altered, which causes a direct local increase in RBC velocity (Wei et al., 2016; Zhou et al., 2019). Currently, our inverse model is parameterized such that vessel diameter changes are the only possible mechanism to regulate blood flow. However, these changes could directly be translated into changes in vessel resistance, which could be translated to changes in ${\tilde{\mu }}_{rel}$. Eventually, these changes of ${\tilde{\mu }}_{rel}$ could be translated to average changes of RBC deformability, given such relation is available.

### 4.3. Inference of Simulation Parameters and Reduction of Network Uncertainties Based on Sparse Experimental Data

Although not extensively studied in this work, we want to highlight that our numerical method can easily be extended for estimating various simulation parameters such as boundary conditions and for reducing overall uncertainties of microvascular networks. This aspect can be highly relevant because of the discussed strong impact of vessel diameter changes on flow rates. Furthermore, the accuracy of blood flow simulations highly depends on the uncertainties related to the acquisition and vectorization of microvascular networks, and on the boundary conditions.

Here we used our method to compare different activation scenarios and the parameter vector α only included the relative diameters of blood vessels. However, Equations (8)–(14) hold for arbitrarily chosen parameters and α could, for example, also include pressure values at boundaries or vessel lengths. Such simulation parameters could be improved by incorporating any other available information into our model, i.e., flow measurements obtained in individual blood vessels or prior knowledge available from literature. Depending on the specific application, a different cost function to Equation (27) would be required.

One drawback of our approach to solve the inverse model is that the adjoint method is intrusive and requires complete knowledge of the underlying physical relations. However, due to its deterministic formulation, we only have to simulate one single realization and therefore, the computational cost is relatively small compared to statistical approaches. This is a clear advantage, if very large networks with many uncertain parameters are considered. For applications, where a deterministic approach is not applicable, using a Bayesian approach as recently proposed by Rasmussen et al. (2017, 2018) could be considered.

### 4.4. Importance of Defining Appropriate Cost Functions

Our numerical method relies on the minimization of a user-defined cost function to solve an inverse problem. The assumptions that are made for defining a cost function have an impact on the simulation result, as demonstrated in Figures 2B,C. Although the desired flow rate increase in *Act* is achieved in both scenarios, the flow distributions outside of *Act* highly depend on the value ρ_*min*_. Similarly, we could also define alternative cost functions, e.g., to minimize the diameter changes in the vasculature or to increase the homogeneity of blood flow. This demonstrates that defining adequate cost functions which represent realistic scenarios is crucial, since the underlying assumptions directly affect the simulation result.

We are confident that the cost function we chose is suitable because our goal was to find the diameters closest to the baseline state which achieve a very localized up-regulation of blood flow. Consequently, we are convinced that our numerical method allows us to obtain meaningful and fundamental results. By comparing various cases where only subsets of blood vessels react, we can evaluate which scenarios are feasible, more likely than others or impossible. Note that the same could also be achieved by manually defining possible combinations of diameter changes of individual vessels and comparing the resulting flow fields. However, our method allows us to do this much more systematically and efficiently, since the target flow distribution is a direct input parameter to the model and the diameter changes are the final simulation result.

We presented a novel numerical method to calculate diameter changes of blood vessels which are needed to achieve localized changes of blood flow in the brain vasculature. The applicability of our method was demonstrated by considering different scenarios in artificial and realistic microvascular networks. In summary, we observed that capillary diameter changes are necessary to obtain a locally confined up-regulation of blood flow. Furthermore, our results revealed that relatively small dilations and constrictions of blood vessels are sufficient to achieve pronounced changes of local blood flow distributions. Of course, many questions regarding the brain's energy supply remain and further studies are necessary to better understand possible regulation mechanisms. Nonetheless, we believe that our numerical method is a convenient tool to systematically investigate the impact of various network and flow parameters on blood flow regulation.

## Data Availability Statement

The original contributions presented in the study are included in the article/Supplementary Materials. Additionally, the raw data is available at: https://doi.org/10.3929/ethz-b-000431445. Further inquiries can be directed to the corresponding author.

## Author Contributions

RE developed the theoretical models, implemented the algorithms, ran the simulations, interpreted the data, and drafted the manuscript. FS and PJ developed the theoretical models and interpreted the results. All authors contributed to the conception of the study and to the revision of the manuscript.

## Conflict of Interest

The authors declare that the research was conducted in the absence of any commercial or financial relationships that could be construed as a potential conflict of interest.
