# Supplementary material for: Predicting Vessel Diameter Changes to Up-Regulate Biphasic Blood Flow During Activation in Realistic Microvascular Networks
Source: Front Physiol. 2020 Oct 16;11:566303. doi: 10.3389/fphys.2020.566303 (PMC7596696; doi:10.3389/fphys.2020.566303)
Supplement: Supplementary file 1 [file Presentation_1.PDF]

Supplementary Material

1 SUPPLEMENTARY EQUATIONS

1.1 Derivation of the discrete adjoint method for blood flow in MVNs.

The goal of our inverse model is to minimize $J(\alpha, \langle p \rangle)$ subject to $\widetilde{g}(\alpha, \langle p \rangle) = 0$. We first define the Lagrangian

$$\mathcal{L}(\alpha, \langle p \rangle, \lambda) := J(\alpha, \langle p \rangle) + \lambda^T \widetilde{g}(\alpha, \langle p \rangle), \tag{S1}$$

where $\lambda \in \mathbb{R}^{N_n}$ is a vector of Lagrange multipliers. Since $\widetilde{g} = 0$ by construction, λ can be any value and $\mathcal{L} = J$, always. The parameter sensitivity of J is

$$\frac{dJ}{d\alpha} = \frac{d\mathcal{L}}{d\alpha} = \frac{\partial J}{\partial \langle p \rangle}\frac{d\langle p \rangle}{d\alpha} + \frac{\partial J}{\partial \alpha} + \frac{d\lambda^T}{d\alpha}\widetilde{g} + \lambda^T \left(\frac{\partial \widetilde{g}}{\partial \langle p \rangle}\frac{d\langle p \rangle}{d\alpha} + \frac{\partial \widetilde{g}}{\partial \alpha} \right), \tag{S2}$$

which can be further simplified and factorized to

$$\frac{dJ}{d\alpha} = \left(\frac{\partial J}{\partial \langle p \rangle} + \lambda^T \frac{\partial \widetilde{g}}{\partial \langle p \rangle} \right) \frac{d\langle p \rangle}{d\alpha} + \lambda^T \frac{\partial \widetilde{g}}{\partial \alpha} + \frac{\partial J}{\partial \alpha}. \tag{S3}$$

For large parameter spaces, computing the term $\frac{d\langle p \rangle}{d\alpha}$ in Eq (S3) is expensive, since it requires $\mathcal{O}(N_n \cdot N_\alpha)$ finite differences. However, we can avoid computing $\frac{d\langle p \rangle}{d\alpha}$ if we calculate λ with the adjoint equation, i.e. Eq (14), which sets the entire first term of Eq (S3) to zero and hence, recovers Eq (13).

2 SUPPLEMENTARY TABLES AND FIGURES

2.1 Tables

Table S1. Average relative diameter changes of different vessel types. The table summarizes the length-averaged diameter changes of arterioles (A), venules (V) and capillaries (C) for all scenarios (I to V). The results were obtained by averaging over the vessels in the entire network, or by only averaging over the vessels within a fixed distance r/r_{act} from the activation centre. A "x" is used to indicate that no vessels of this type are present in the corresponding averaging region. Values are relative changes in percent.

	**Entire network**			$\mathbf{r/r_{act} < 2}$			$\mathbf{r/r_{act} < 1}$		
	A	V	C	A	V	C	A	V	C
**I**	0.08	0.14	0.06	0.84	3.34	0.72	1.59	x	2.75
**II**	0.12	0.00	0.09	1.24	0.00	0.95	2.30	x	3.50
**III**	0.00	0.00	0.16	0.00	0.00	1.42	0.00	x	5.18
**IV**	0.57	0.59	0.00	4.49	6.28	0.00	7.71	x	0.00
**V**	0.81	0.00	0.00	6.58	0.00	0.00	10.95	x	0.00

Table S2. Relative changes of blood flow in different vessel types. The table summarizes relative changes of length-weighted total flow rates in arterioles (A), venules (V) and capillaries (C) for all scenarios (I to V). The results were obtained by considering the total flow rates in vessels in the entire network, or by only considering the vessels within a fixed distance r/r_{act} from the activation centre. A "x" is used to indicate that no vessels of this type are present in the corresponding region. Values are relative changes in percent.

	**All**			**r/ract<2**			**r/ract<1**		
	A	V	C	A	V	C	A	V	C
**I**	-0.05	0.22	0.17	0.14	9.77	4.78	-0.29	x	30.02
**II**	0.09	0.17	0.33	0.61	4.74	4.39	-0.17	x	30.03
**III**	0.02	0.07	0.16	0.09	2.61	3.09	0.23	x	29.71
**IV**	2.39	0.99	2.11	10.48	17.31	14.04	4.44	x	30.04
**V**	3.63	2.23	3.71	14.84	6.71	16.77	2.59	x	29.98

2.2 Figures

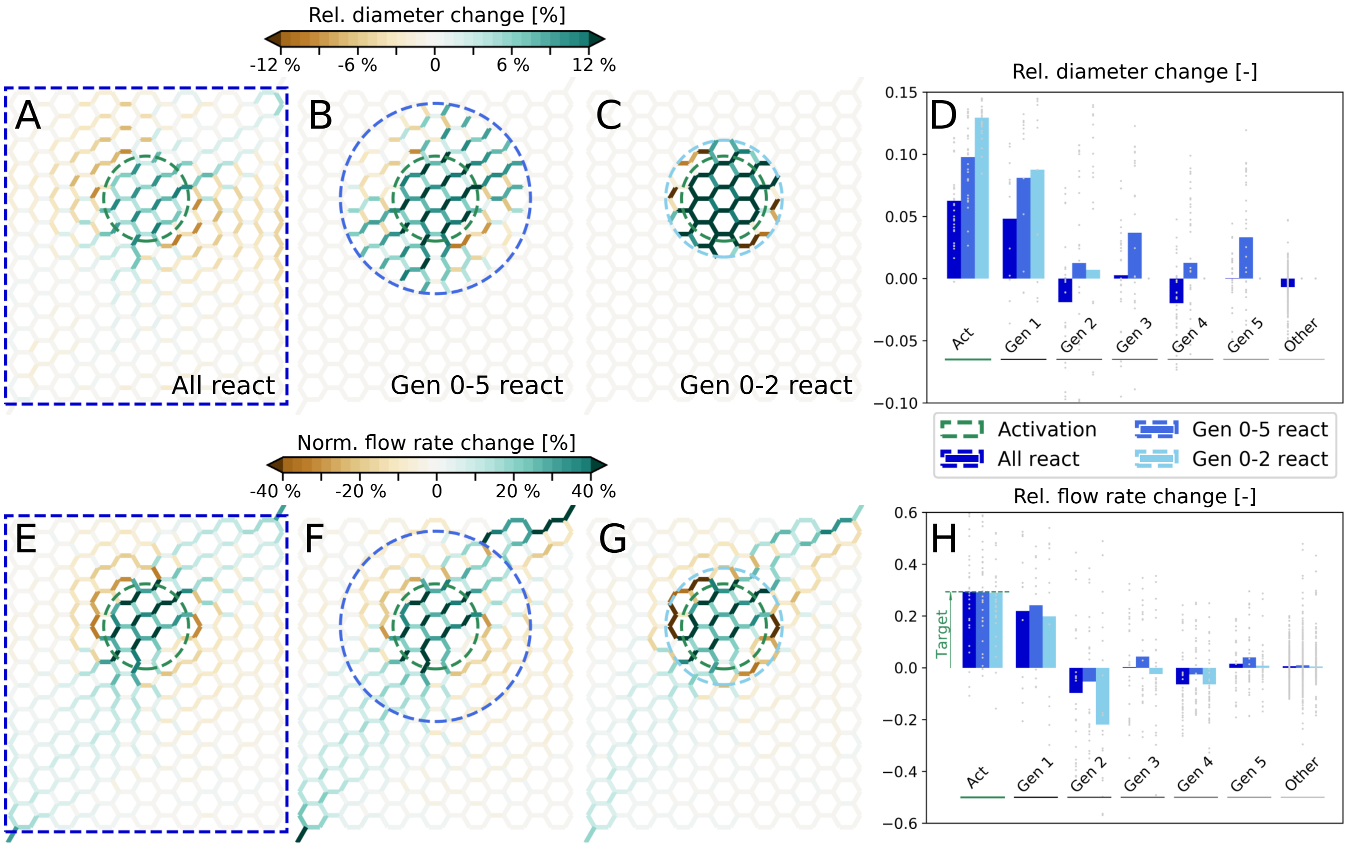

Figure S1. Supporting figure to demonstrate that the results of the inverse model are robust with respect to the chosen bifurcation rule. Instead of applying a bifurcation rule based on empirical data (Figure 4), here, the RBCs always follow the path of the largest pressure force and therefore, are always assigned to the daughter vessel with the largest bulk flow velocity. Note that the differences to Figure 4 are small.

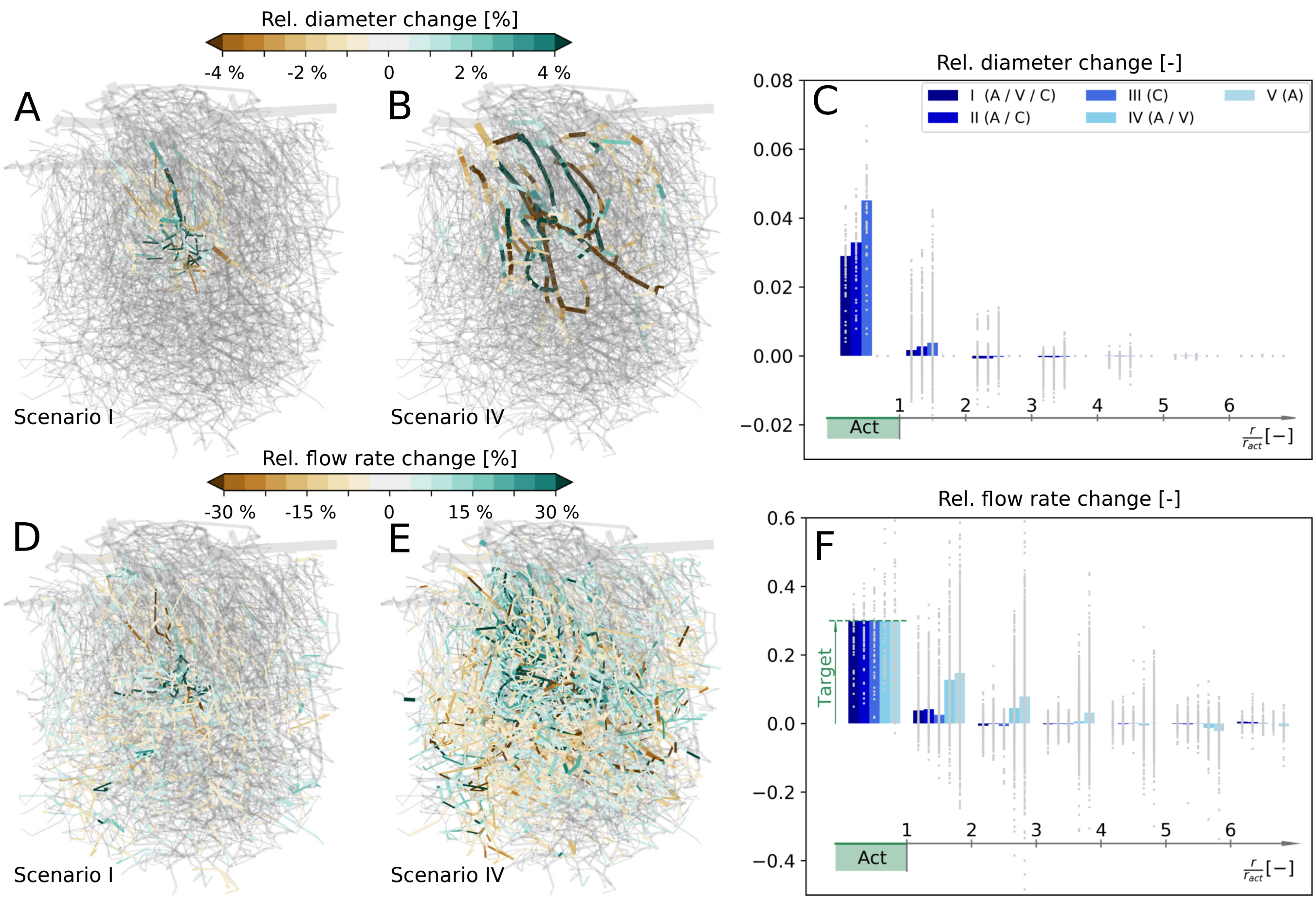

Figure S2. Supporting figure to demonstrate that the results of the inverse model are robust with respect to the chosen bifurcation rule. Instead of applying a bifurcation rule based on empirical data (Figure 5), here, the RBCs in blood vessels with $d \leq 6\,\mu\text{m}$ always follow the path of the largest pressure force. In larger vessels, RBCs are assigned to daughter vessels proportional to the corresponding fractional blood flow rates. Note that the differences to Figure 5 are small.

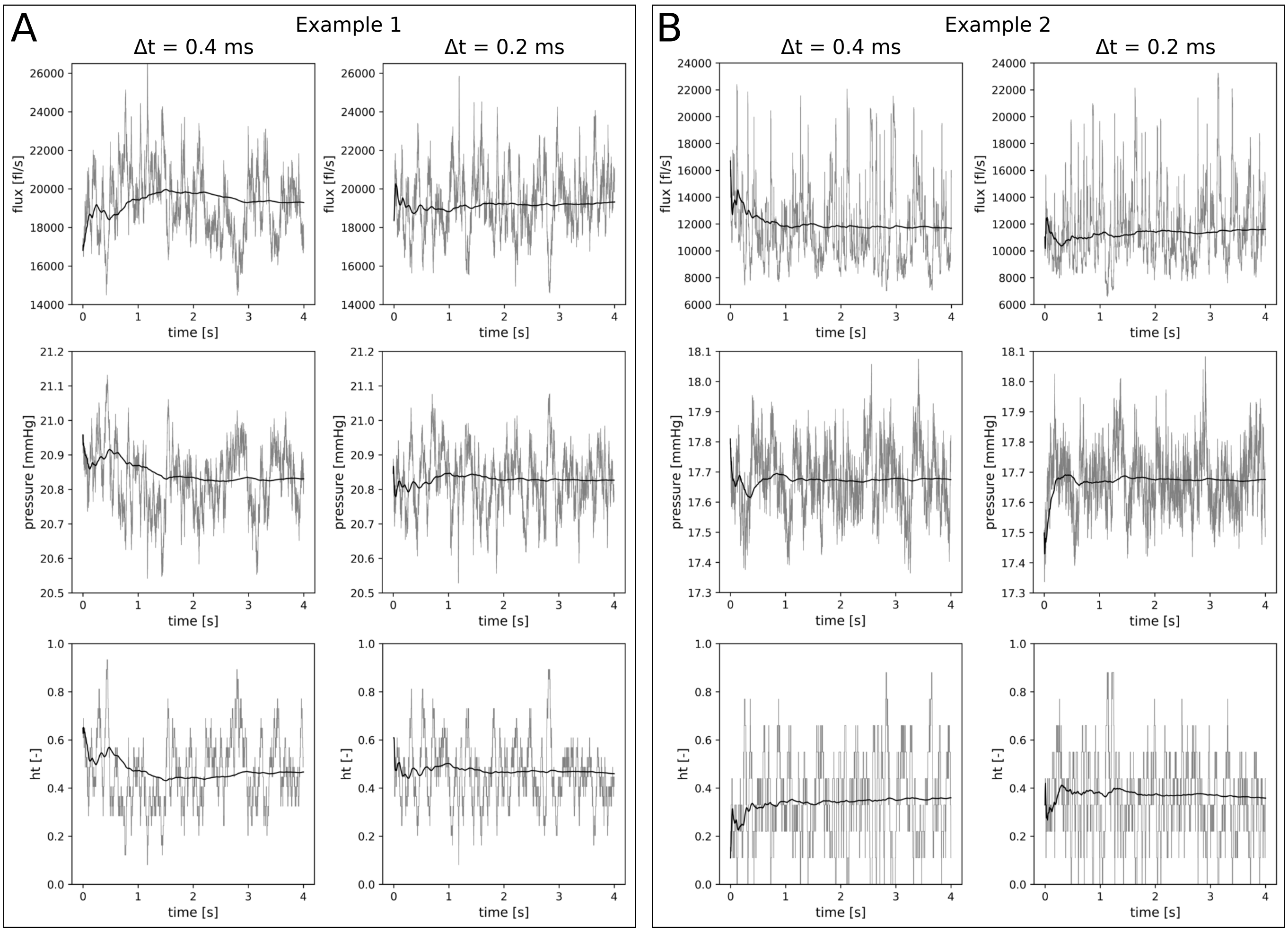

Figure S3. Convergence of time averaged flow rates, pressures and haematocrit, shown on the examples of two capillaries (**A+B**) which are located in the activated region of the realistic MVN. Grey lines denote instantaneous quantities at any given time and black lines are the values obtained by averaging over all previous time steps. Note that in our inverse model, only the time averaged flow rates and pressures are required. For both examples, the results for two different fixed time step sizes are given, i.e. $\Delta t = 0.4\,\mathrm{m\,sec}$ and $\Delta t = 0.2\,\mathrm{m\,sec}$. The instantaneous values are highly heterogeneous and can change suddenly. After an averaging time of 4 sec, the averaged quantities have reached approximately statistically steady state values. It is important to note that the prescribed flow changes in the activation scenario are larger than the fluctuations in the averaged simulation results, which suggests that an averaging interval of 4 sec is a suitable choice. Furthermore, comparable averaged values are obtained for $\Delta t = 0.4\,\mathrm{m\,sec}$ and $\Delta t = 0.2\,\mathrm{m\,sec}$. For a time step size $\Delta t = 0.4\,\mathrm{m\,sec}$, bifurcation events occur on average in 3.3 % of all capillaries. Moreover, the average number of newly assigned RBCs per time step is less than one in 99.9 % of all capillaries. Consequently, the number of bifurcation events per time step in the capillary bed is relatively low. Note that for the most accurate result, an adaptive time step size should be chosen such that only one bifurcation event takes place per time step. However, computational cost would be unfeasibly high for large networks, where many bifurcation events take place almost simultaneously.

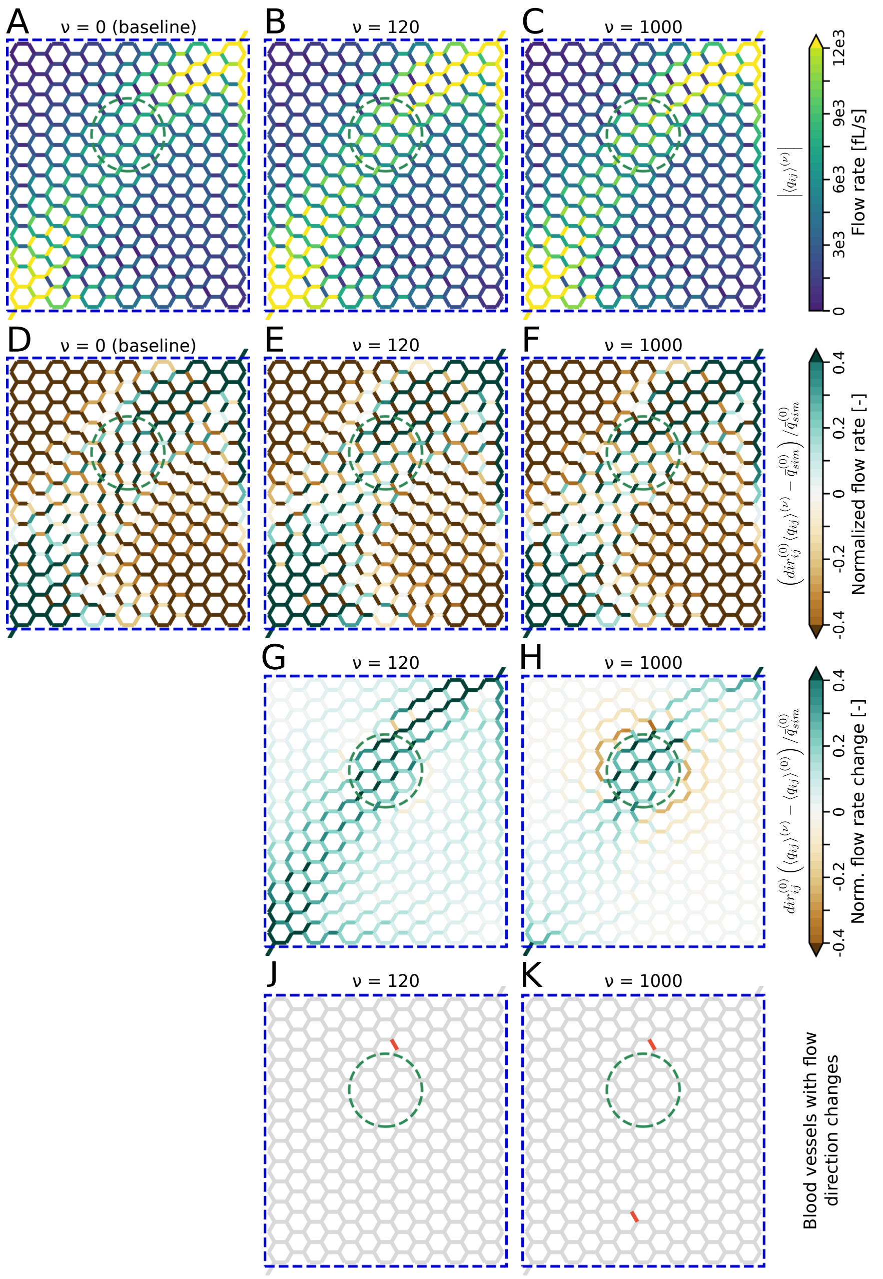

Figure S4. Supporting figure to Figure 2 to demonstrate how the normalized flow rate changes during activation were calculated based on the absolute flow rate distributions. **(A)** Absolute flow rates at baseline ($\nu = 0$). Note that the colourbar is limited to the range from 0 to $12\,000\,\mathrm{fL\,sec^{-1}}$ and the flow rate over the boundaries is $67\,375\,\mathrm{fL\,sec^{-1}}$. **(B)** Absolute flow rates during activation without secondary constraints ($\nu = 120$). The flow rate over the boundaries is $78\,348\,\mathrm{fL\,sec^{-1}}$. **(C)** Absolute flow rates during activation with secondary constraints ($\nu = 1000$). The flow rate over the boundaries is $70\,017\,\mathrm{fL\,sec^{-1}}$. **(D-F)** Normalized flow rates for $\nu = 0$, $\nu = 120$ and $\nu = 1000$. The flow rates are normalized with the mean flow rate in the activated region at baseline, i.e. $\bar{q}_{sim}^{(0)}$. Therefore, green and brown colours correspond to flow rates which are higher and lower, if compared to $\bar{q}_{sim}^{(0)}$. **(G,H)** Flow rate changes at $\nu = 120$ and $\nu = 1000$, normalized with $\bar{q}_{sim}^{(0)}$. The two figures were obtained by subtracting the baseline flow distributions **(D)** from the distributions at $\nu = 120$ **(E)** and $\nu = 1000$ **(F)**, respectively. Note that these two figures **(G,H)** are identical to Figures 2B and C. **(J,K)** Red colour is used to highlight the blood vessels in which the flow directions change during activation for $\nu = 120$ and $\nu = 1000$, respectively.

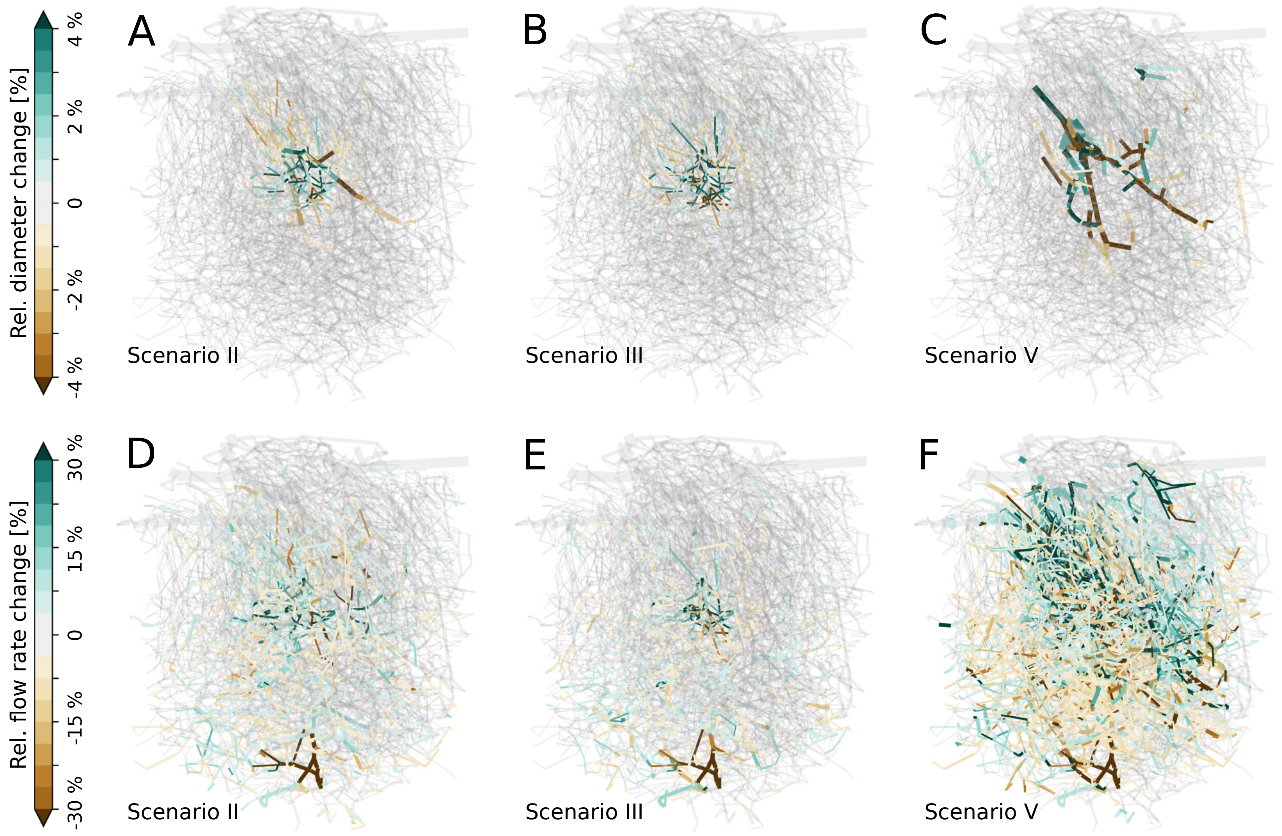

Figure S5. Supporting figure to Figure 5: Relative diameter and flow rate changes in a realistic MVN for scenarios II, III and V. **(A)** Diameter changes if A and C can react (scenario II). **(B)** Diameter changes if only C can react (scenario III). **(C)** Diameter changes if only A can react (scenario V). **(D-F)** Corresponding blood flow changes.

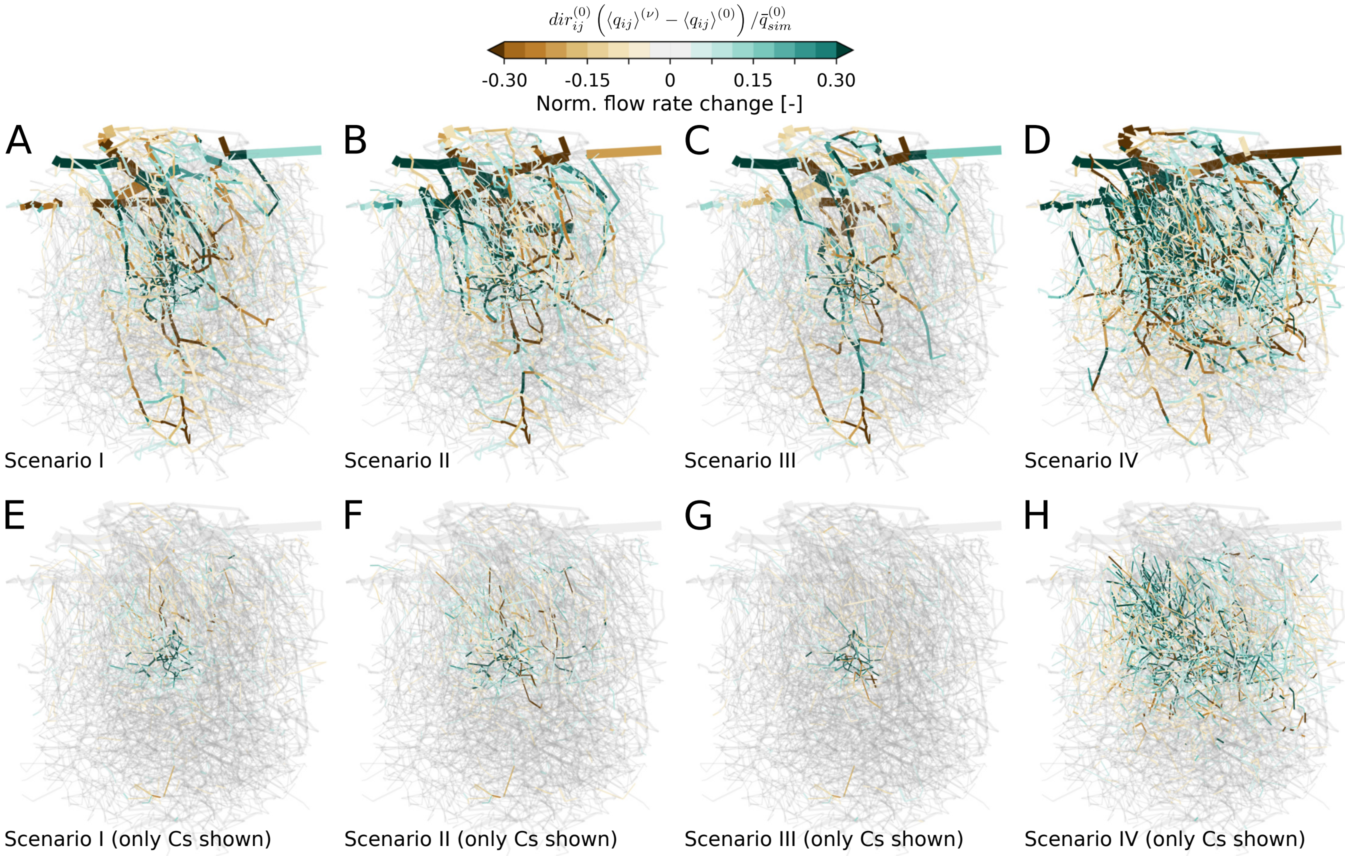

Figure S6. Supporting figure to Figure 5: Normalized flow rate changes in a realistic microvascular network during activation for scenarios I-IV. **(A)** Normalized flow rate changes in all blood vessels during activation for scenario I. The absolute flow rate changes are normalized with the mean capillary blood flow rate in the activated barrel at baseline, i.e. $\bar{q}_{sim}^{(0)}$, which is consistent with the results shown in Figures 4E - G for the artificial hexagonal network. Note that the normalized flow rate changes can be high in large blood vessels, i.e. in PA, PV, A and V. This is due to the heterogeneity of flow rates in MVNs, where the blood flow in PA, PV, A and V can be orders of magnitude higher than in C. Therefore, the normalization with the relatively small $\bar{q}_{sim}^{(0)}$ leads to substantial normalized changes, although the corresponding relative flow rate changes are small (Figure 5D). Similarly, moderate absolute flow rate changes can lead to very high relative changes if the baseline flow rate is low, i.e. close to zero. This has to be kept in mind while visualizing and interpreting results for individual blood vessels in realistic MVNs and is one of the reasons that averaging cubes were used in Figure 5F. **(B-D)** Normalized flow rate changes in all blood vessels for scenarios II-IV. **(E-H)** Normalized flow rate changes in capillaries. These figures are identical to **A-D**, but the changes in PA, PV, A and V are hidden and only the changes in C are shown. For scenarios I-III, the blood flow increase is much more confined to *Act* than for scenario IV, where it also spreads into neighbouring barrels.

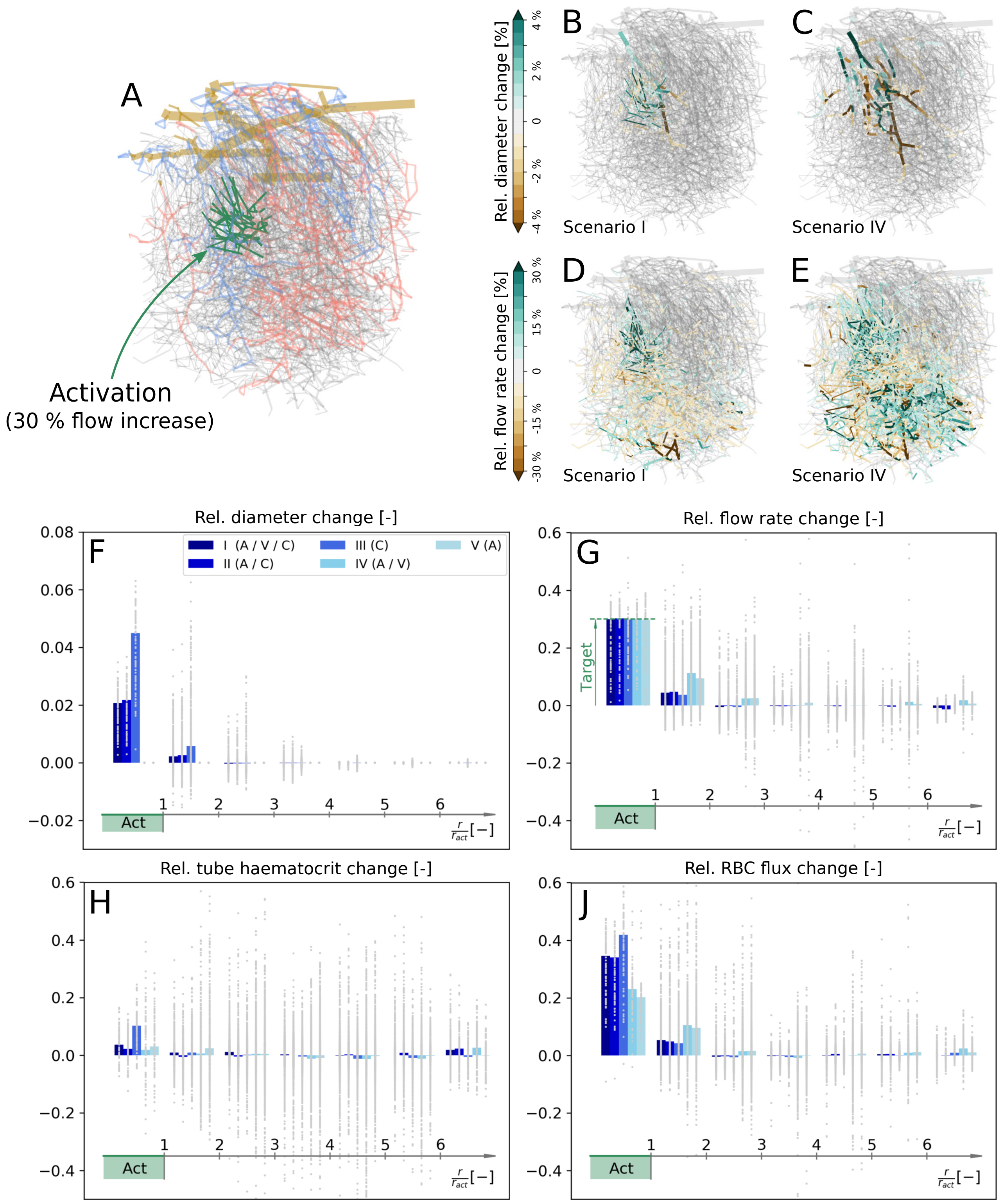

Figure S7. Additional results for a test case where the activated region was moved to a barrel located next to the barrel analysed in the results section of the paper (Figures 5 and 6). **(A)** Visualization of the activated region. **(B+C)** Relative diameter changes for scenarios I and IV. **(D+E)** Corresponding relative changes of blood flow rates. **(F-J)** Averaged relative changes of diameter, flow rate, tube haematocrit and RBC flux as functions of distance to the barrel centre.

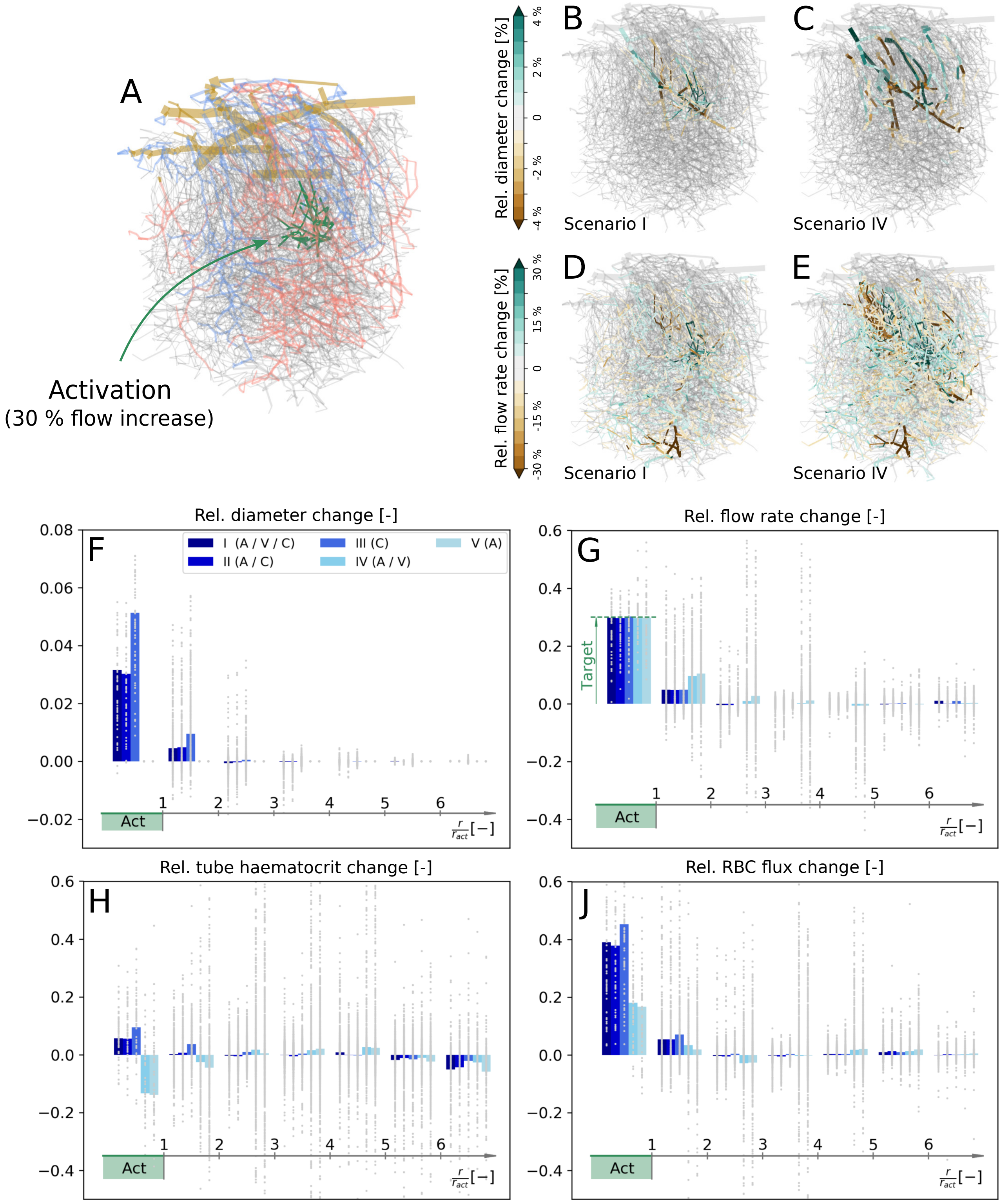

Figure S8. Additional results for a test case where the activated region was moved to a barrel located next to the barrel analysed in the results section of the paper (Figures 5 and 6). **(A)** Visualization of the activated region. **(B+C)** Relative diameter changes for scenarios I and IV. **(D+E)** Corresponding relative changes of blood flow rates. **(F-J)** Averaged relative changes of diameter, flow rate, tube haematocrit and RBC flux as functions of distance to the barrel centre.
